# Role of immigration and emigration on the spread of COVID-19 in a multipatch environment: a case study of India

**DOI:** 10.1038/s41598-023-37192-z

**Published:** 2023-06-29

**Authors:** Tanuja Das, Shraddha Ramdas Bandekar, Akhil Kumar Srivastav, Prashant K Srivastava, Mini Ghosh

**Affiliations:** 1 Department of Mathematics and Statistics, University of New Brunswick, Fredericton, Canada; 2 Department of Integrative Biology, University of Texas at Austin, Austin, TX USA; 3 Mathematical and Theoretical Biology, BCAM - Basque Center for Applied Mathematics, Bilbao, Spain; 4 Department of Mathematics, Indian Institute of Technology Patna, Patna, 801103 India; 5 Division of Mathematics, School of Advanced Sciences, Vellore Institute of Technology, Chennai, India

**Keywords:** Mathematics and computing, Infectious diseases

## Abstract

Human mobility has played a critical role in the spread of COVID-19. The understanding of mobility helps in getting information on the acceleration or control of the spread of disease. The COVID-19 virus has been spreading among several locations despite all the best efforts related to its isolation. To comprehend this, a multi-patch mathematical model of COVID-19 is proposed and analysed in this work, where-in limited medical resources, quarantining, and inhibitory behaviour of healthy individuals are incorporated into the model. Furthermore, as an example, the impact of mobility in a three-patch model is studied considering the three worst-hit states of India, i.e. Kerala, Maharashtra and Tamil Nadu, as three patches. Key parameters and the basic reproduction number are estimated from the available data. Through results and analyses, it is seen that Kerala has a higher effective contact rate and has the highest prevalence. Moreover, if Kerala is isolated from Maharashtra or Tamil Nadu, the number of active cases will increase in Kerala but reduce in the other two states. Our findings indicate that the number of active cases will decrease in the high prevalence state and increase in the lower prevalence states if the emigration rate is higher than the immigration rate in the high prevalence state. Overall, proper travel restrictions are to be implemented to reduce or control the spread of disease from the high-prevalence state to other states with lower prevalence rates.

## Introduction

The pattern and spread of diseases such as COVID-19 may vary from place to place in a vast country like India, which is divided geographically into many states. Many factors may contribute to such irregular patterns among these different states. These factors include travel, population density, literacy, community behaviour, healthcare infrastructure, etc. Hence, the spread in different states may follow a different pattern. Therefore, in this study, we propose a multi-patch COVID-19 model to address these concerns and study the impacts of individuals’ mobility along with other important factors such as limited medical resources and inhibitory behaviour of individuals due to the disease prevalence.

The novel Coronavirus SARS-CoV-2 first emerged in Wuhan, China^1^ and has spread worldwide, and become a fatal pandemic. As per WHO^2^, this disease spreads via nasal discharge and saliva droplets when one sneezes or coughs. It is spreading in an unforeseen way in various parts of the world, and different variants of the same have been reported since then. Whether a country is developed, developing, or poor, the cases have been rising and declining in an unprecedented way. This could be due to several reasons like the mobility of individuals, low vaccination, improper testing drives, no compulsion to practice of social distancing or to wear face masks, no governmental interventions, lack of medical resources and political disputes, etc. Travel plays a key role in helping the spread of infectious diseases by the introduction of a pathogen in a new area. In the study by^3^, travel or migration is emphasized to be the potential force in disseminating infectious diseases. With an ongoing pandemic that spreads through direct contact and where hygiene plays a significant role, frequent movement of individuals from a higher prevalence area to a remote novel village or town with minimal prevalence can increase the risk of viral spillover. Considering the worst hit states of India, which are Kerala, Maharashtra and Tamil Nadu summing up to a population of more than 217 million, human mobility would certainly contribute to accelerating the spread, as individuals carrying the virus have different genetic makeup and varied immunological experiences. Looking into the aspect of the rate at which the virus traveled the world and impacted the entire human livelihood, the study in^4^ covered the impact of mobility restrictions on disease spread, in the most populous nation, China. Their findings suggested that there could be a possible delay in epidemic peak provided two regions with higher risks were locked down.

Various studies on the mathematical modeling of COVID-19 have been conducted with various aspects since the beginning of the pandemic. Most of these works are based on the classic compartmental model^5^. A brief literature review on different *SEIR* models and other extended compartmental models, as well as the COVID-19 disease origin, COVID-19 transmission with and without interventions, has been discussed by Anirudh^6^ and Meehan et al.^7^. Working on a network model, it is shown that the disease transmission rate of COVID-19 is higher than SARS^8^. To estimate the unreported infective in China, an *SEIR* model with unreported infective has been proposed^9^. Bandekar and Ghosh developed a compartmental model with reported and unreported cases along with quarantine and hospitalization to estimate infective detection rates for India and three of its states^10^. Some interventions are found to be of prime importance in studying these compartmental models. In^11^, the authors worked on SEIRD model and developed the system by means of partial differential equations coupled with a heterogeneous diffusion model. A very interesting study in^12^ presents the co-infection dynamics of Zika and SARS-CoV-2 capturing sexual transmission of Zika in the model. It further shows that the inclusion of time-dependent controls pertaining to Zika and COVID-19 mitigation strategies could significantly reduce the co-infection burden. In a similar context, the study by^13^ deals with HBV and COVID-19 co-infection dynamics with optimal control.

Forecasting disease spread is emphasized to identify various determinants and effectively implement intervention strategies^14^. Authors emphasized the importance of face masks, sanitation, social distancing, and other non-pharmaceutical interventions to combat the spread of the disease^15–18^. Similarly, Srivastav et al. provided predictions on the disease dynamics considering face-mask efficacy, quarantine, and hospitalization facilities^19^. Along with these interventions, the availability of adequate medical resources is a significant concern in pandemic-affected countries. The number of cases is increasing, and there are insufficient beds, ventilators, and oxygen cylinders to accommodate the infected individuals. In addition, the front-line workers are struggling due to the lack of an adequate number of PPE kits^20^. As per report^21^, India had one bed per 2000 individuals before the epidemic hit the country. A detailed study based on the limitation of medical resources is presented for India, Brazil, and the USA, with an analysis on backward bifurcation when the number of infective exceeds a threshold value^22^. Therefore, quarantine, precautionary measures, and a limited treatment facility are three crucial factors affecting the prevalence of COVID-19 disease. In the study^23^ also impacts of several non-pharmaceutical measures on the population dynamics of COVID-19 in Nigeria is analysed. Their findings suggested that a detection rate of 0.8 per day with approximately 55% population abiding by social distancing norms could lead to a significant decrease in new cases and hence the prevalence of the disease.

In this study, a multi-patch model is developed for COVID-19. Several studies for various other infectious diseases have been conducted using the multi-patch models^8,24–27^. In^24^, the authors have worked on a multi-patch dengue model depicting spatial and temporal transmission and effectiveness of mosquito control strategies in Kolkata, India. The study concluded that controls exhibiting higher environment persistence performed better and connectedness between different regions makes control strategies effective. In^25^, Hsieh et al. proposed a multi-patch model to study the impact of people’s mobility during the epidemic of influenza spreading while different patches had different infection levels. In a similar context, the authors in the study^26^ worked on a multi-patch dengue model focusing on the influence of the Wolbachia bacterium in the optimal control study. The impact of mobility on the spatial spread of dog rabies between two patches involving different disease prevalence levels was studied by Liu et al. in^27^, by developing a two-patch *SEIRS* compartmental model. The significance of short-term human mobility is studied by framing a theoretical two-patch model in^28^. Additionally, a cost-effective strategy and optimal control analysis was applied to provide better insights into the control of disease spread considering economic aspects as well. The impact of migration on infectious diseases involving two patch systems^29^ and *n*-patch system^30^ is performed to show the mobility impact on spatial distribution. In a multi-patch, flux-based model, the geometry of migrations between patches is determined along with the influx of the infective into a patch^31^. The study shows the effect of time delay between the influx of the infective into a patch and the basic reproduction number. The global stability of a multi-group *SIR* and a delayed multi-group *SIS* model with patch structure is investigated^32,33^. These studies conclude that migration and incidence delays do not affect disease dynamics. The study by Zhang et al. focused on a multi-patch epidemic model with distributed delays considering both deterministic and stochastic scenarios^34^. They used nonlinear incidence rates and considered the waning immunity of the recovered population. The dynamics of a susceptible-infected-susceptible model along with a detailed analysis of the asymmetric connectivity matrix associated with the model has been studied^35^. According to the study, the basic reproduction number decreases as the infection spreads. The sufficient conditions for disease extinction and persistence have been established by working on a two-patch *SIR* model with varying population sizes and regime switching^36^. The investigation of COVID-19 transmission in a multi-patch environment is interesting and crucial to draw significant conclusions about the impact of mobility on disease spread. However, there has not been much progress in this direction. Meng et al. investigated a multi-patch *SEIR* compartmental model of COVID-19 disease spread^37^. They assumed a three-patch model to numerically explain the effect of migration and quarantine rate on the basic reproduction number.

In this study, we consider a multi-patch model which accounts for the quarantine, saturated treatment, and nonlinear incidence rate. The nonlinear incidence rate accounts for the inhibitory behaviour of healthy individuals to avoid contracting the disease. The inter-patch movement is allowed in susceptible, exposed, asymptomatic, and recovered compartments only. Individuals who are symptomatic, under medical supervision, or have been quarantined are presumed unable to move. The main purpose of this study is to understand the effect of the movement of populations and its impact on the spread of COVID-19. As the movement is also impacted by various other factors during COVID-19, we shall consider specific cases of movement of populations in this work. We have also accounted for the saturated treatment in the model reflecting the limitation of the medical resources during the disease outbreak. A practical example is exhibited numerically by a particular 3-patch model and performing data fitting and predictive analysis for that. The paper is organized as follows. In the following section, a multi-patch model is proposed and analysed. The existence and stability of disease-free equilibrium and endemic equilibrium are examined. In sections “results”, “parameter estimation and short time prediction”, “discussion”, we explore three different cases: when both emigration and immigration are allowed when only emigration is allowed, and when neither emigration nor immigration is allowed. Section “conclusion” is devoted to estimating parameters using data from three states of India, Kerala, Tamil Nadu, and Maharashtra, as an example. The short-term and long-term predictions are provided in the following section. In the end, we summarize the results and conclude our work.

## The *n*-patch *SEIQHR* model for COVID-19

As discussed in the introduction, we consider an *n*-patch model for COVID-19. Assume that the total population is divided into *n*- patches. Further, in each patch, the population is subdivided into seven compartments: susceptible, exposed, asymptomatic, quarantined, symptomatic, hospitalized or home isolated, and recovered. Therefore, we choose a metapopulation system consisting of arbitrary *n*- patches where $S_i$, $E_i$, $I_{a_i}$, $Q_i$, $I_{s_i}$, $H_i$, and $R_i$ are corresponding compartments in the *i*-th patch (see Table 1). Hence the total population *N* is the sum of individuals in all *n*-patches, i.e., $N=\sum _{i=1}^n (S_i+E_i+I_{a_i}+Q_i+I_{s_i}+H_i+R_i)=\sum _{i=1}^n N_i$.

**Table 1:** Description of variables considered in the model system (1).

Variable	Description
$S_i$	Compartment of susceptible individuals in the *i*-th patch
$E_i$	Compartment of exposed individuals in the *i*-th patch
$I_{a_i}$	Compartment of asymptomatic individuals in the *i*-th patch
$Q_i$	Compartment of quarantined individuals in the *i*-th patch
$I_{s_i}$	Compartment of symptomatic individuals in the *i*-th patch
$H_i$	Compartment of hospitalized or home isolated individuals under medical supervision in the *i*-th patch
$R_i$	Compartment of recovered individuals in the *i*-th patch
$N_i$	Total population in the *i*-th patch

In the following, we shall describe each compartment in the *i*-th patch along with the relevant assumptions for developing the model (1).

1. All newborns are assumed to be susceptible and enter the susceptible compartment $S_i$ with a constant birth rate $\Lambda _i$. Susceptible individuals interact with COVID-19 infected individuals, thereby becoming exposed and leaving the susceptible compartment and joining the exposed compartment. As is the case for COVID-19, an infected individual may or may not exhibit symptoms. So, both symptomatic and asymptomatic can transmit the infection. Information about the spread and prevalence of the disease has an inhibitory effect on the healthy population. Hence, the interaction term is modeled as a nonlinear saturated type function, similar to the one studied by Capasso and Serio^38^ accounting for this inhibitory behaviour. The interaction between susceptible and symptomatic individuals occurs in each patch in the form, $\frac{\beta _{s_i}}{1+m_{s_i} I_{s_i}}I_{s_i}$ which is saturated by $\frac{\beta _{s_i}}{m_{s_i}}$ due to the effect of inhibition. Similarly, the interaction between susceptible and asymptomatic individuals also occurs in each patch in the form, $\frac{\beta _{a_i}}{1+m_{a_i} I_{a_i}}I_{a_i}$ which is saturated by $\frac{\beta _{a_i}}{m_{a_i}}$ due to the effect of inhibition. Here $\beta _{s_i}$ and $\beta _{a_i}$ are infection rates corresponding to symptomatic and asymptomatic populations. Here $m_{s_i}$ and $m_{a_i}$ are constants representing inhibitory effects in symptomatic and asymptomatic populations, respectively, due to information in the population.
2. Individuals in exposed compartment $E_i$ are exposed to infection but are yet to be infectious. Within two weeks (on average ten days for COVID-19^39^) after exposure, the exposed one becomes infectious and leaves the exposed compartment.
3. Individuals in the asymptomatic compartment $I _{a_i}$ are infected but do not exhibit symptoms. They are identified through contact tracing^40^. It is assumed that a fraction of these will be quarantined based on information if they come in contact with an infected individual, and those who exhibit symptoms in this compartment will move to the symptomatic compartment.
4. Individuals in quarantined compartment $Q_i$ are assumed to remain in quarantine and thus do not spread disease. If they develop symptoms during quarantine, they can either join the symptomatic compartment or recover and move to the recovered compartment.
5. Individuals who start showing symptoms move to the symptomatic compartment $I_{s_i}$ from the exposed compartment. According to the government advisory, symptomatic infected individuals must be either self-isolated at home or hospitalized. So, individuals leave this compartment either via hospitalization and receiving medical attention, which is considered saturated to emphasize that all individuals seeking medical attention may not receive it^41^ or by going into self-isolation at a rate of $\alpha _{s_i} I_{s_i}$. As mentioned above, this compartment receives inflow from $E_i$, $I_{a_i}$, and $Q_i$.
6. Individuals in compartment $H_i$ are either hospitalized and receiving medical attention or in home isolation and may or may not be receiving medical attention. As these individuals are under observation, they are isolated from the rest of the population and cannot spread infection. They either recover and join the recovered compartment or die due to disease infection.
7. The last compartment, $R_i$ contains recovered individuals. These individuals are assumed to have short-term immunity and thus cannot be infected again during the modeling period.
8. It is assumed that the movement from one patch to another is allowed in compartments $S_i$, $E_i$, $I_{a_i}$ and $R_i$ only. We denote $a_{lk}$, $b_{lk}$, $c_{lk}$ and $d_{lk}$ as the traveling rates from patch *k* to patch *l* of susceptible, exposed, asymptomatic, and recovered individuals, respectively. Individuals in other compartments i.e., $I_{s_i}$ and $H_i$, cannot move as they are severely infected.

The following table summarizes all the parameters considered in patch *i*, described above for each population compartment (Table 2).

**Table 2:** Explanation of parameters included in *i*th patch of system (1).

Parameters	Description	Values
$\Lambda _i$	Constant recruitment rate of individuals in susceptible compartment	Vary
$\beta _{s_i}$	Effective contact rate between susceptible and symptomatic individuals	Estimated
$\beta _{a_i}$	Effective contact rate between susceptible and asymptomatic individuals	0.00005–0.0002
$m_{s_i}$	Rate constant representing inhibitory constant for symptomatic individuals	0.1
$m_{a_i}$	Rate constant representing inhibitory constant for asymptomatic individuals	0.1
$\mu _i$	Natural mortality rate of individuals	0.003
$\sigma _i$	Disease infection rate	0.16^19^
$\gamma _i$	Quarantining rate of asymptomatic individuals	Estimated
$\theta _i$	Rate of showing symptom in asymptomatic individuals	0.01–0.08^19,42^
$\eta _i$	Rate of showing symptom in quarantined individuals	0.001
$\alpha _{s_i}$	Isolation rate of symptomatic individuals	Estimated
$\alpha _i$	Hospitalization rate of symptomatic individuals	0.02–0.1^19^
$\nu _i$	Saturation constant representing limitation of medical resources	0.01
$\mu _{s_i}$	Disease mortality rate of symptomatic individuals	0.0052^19^
$\mu _{h_i}$	Disease mortality rate of hospitalised individuals	0.0042^19^
$\rho _i$	Recovery rate of self-quarantined individuals	0.0002–0.002^19,43^
$\xi _i$	Recovery rate of hospitalised/self-isolated individuals	$\frac{1}{14}$^1,19^
$\varepsilon _i$	Proportion of exposed individuals not showing clinical symptoms	0.07–0.33^19,39^

**Figure 1:**
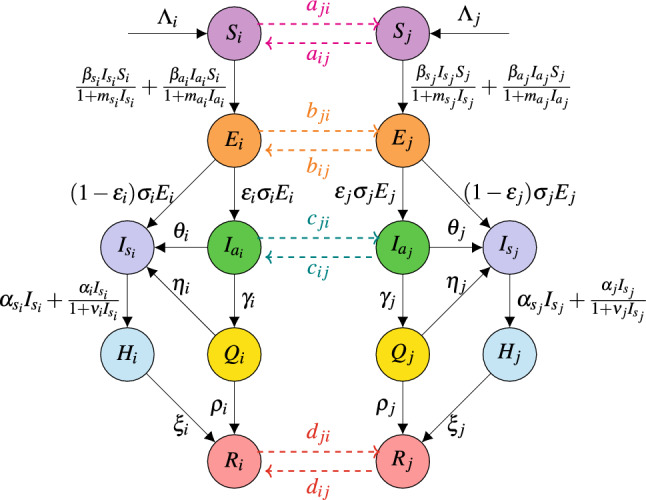
Flow diagram of the multi-patch model (1) showing interactions between patch *i* and patch *j*.

The inter and intra patch interactions and mobility of populations in patch *i* and patch *j* are represented by Fig. 1.

Mathematically, we express the interactions of individuals in patch *i* with other patches, $j= 1,2,\ldots ,n$ such that $j\ne i$ with the help of the following system of differential equations. Note that this representation is true for all $n-$ patches (i.e. for each *i*th patch with $i=1,2,\ldots ,n$).

1 $$\begin{aligned} \begin{aligned} \frac{dS_i}{dt}&=\Lambda _i-\mu _i S_i-\frac{\beta _{s_i}}{1+m_{s_i} I_{s_i}}S_iI_{s_i}-\frac{\beta _{a_i}}{1+m_{a_i}I_{a_i}}S_iI_{a_i}+\sum _{\begin{array}{c} j=1,\\ j\ne i \end{array}}^{n}(a_{ij}S_j-a_{ji}S_i),\\ \frac{dE_i}{dt}&=\frac{\beta _{s_i}}{1+m_{s_i} I_{s_i}}S_iI_{s_i}+\frac{\beta _{a_i}}{1+m_{a_i}I_{a_i}}S_iI_{a_i}-(\sigma _i+\mu _i) E_i+\sum _{\begin{array}{c} j=1,\\ j\ne i \end{array}}^{n}(b_{ij}E_j-b_{ji}E_i), \\ \frac{dI_{a_i}}{dt}&= \varepsilon _i\sigma _i E_i-\mu _iI_{a_i}-\gamma _iI_{a_i}-\theta _i I_{a_i}+\sum _{\begin{array}{c} j=1,\\ j\ne i \end{array}}^{n}(c_{ij}I_{a_j}-c_{ji}I_{a_i}),\\ \frac{dQ_i}{dt}&= \gamma _iI_{a_i}-(\eta _i+\rho _i+\mu _i) Q_i, \\ \frac{dI_{s_i}}{dt}&=(1-\varepsilon _i)\sigma _i E_i-(\mu _{s_i}+\mu _i)I_{s_i}+\eta _iQ_i+\theta _i I_{a_i}-\alpha _{s_i} I_{s_i}-\frac{\alpha _i I_{s_i}}{1+\nu _i I_{s_i}},\\ \frac{dH_i}{dt}&=\alpha _{s_i} I_{s_i}+\frac{\alpha _i I_{s_i}}{1+\nu _i I_{s_i}}-(\mu _{h_i}+\mu _i) H_i-\xi _i H_i,\\ \frac{dR_i}{dt}&= \rho _iQ_i+\xi _i H_i-\mu _i R_i+\sum _{\begin{array}{c} j=1,\\ j\ne i \end{array}}^{n}(d_{ij}R_j-d_{ji}R_i). \end{aligned} \end{aligned}$$

We denote the mobility matrices as $A=[a_{ij}]_{n\times n}$, $B=[b_{ij}]_{n\times n}$, $C=[c_{ij}]_{n\times n}$, $D=[d_{ij}]_{n\times n}$ with $a_{ii}=b_{ii}=c_{ii}=d_{ii}=0$ $\forall\, i=1,2,\ldots ,n$.

### Theorem 2.1

Denote, $${\bar{\Lambda }}=\sum _{i=1}^n \Lambda _i$$ and $${\bar{\mu }}=\min \{ \mu _i \}$$, $$i=1,2,\ldots ,n$$.

The basic feasible region of system (1) is given by,

$$\begin{aligned} \Gamma := \{(S_1,E_1,I_{a_1},Q_1,I_{s_1},H_1,R_1,\ldots ,S_n,E_n,I_{a_n},Q_n,I_{s_n},H_n,R_n)\in {\mathbb {R}}^{7n}_+| \; 0\le N(t)\le \frac{{\bar{\Lambda }}}{{\bar{\mu }}}, 0\le S_i\le S_{0_i}, i=1,2,\ldots ,n\}, \end{aligned}$$

where $S_{0_i}$ is defined in the proof.

### Proof

Consider the system (1) with all non negative initial values holding $N_i(0)\ge 0, i=1,2,\ldots ,n$. It can be easily verified using similar arguments as described by Srivastav et al.^19^ that all the compartments in each patch remain non negative for all positive time if the initial values lie in ${\mathbb {R}}^{7n}_+$ .

Further, from model (1) the total population *N*(*t*) satisfies,

$$\begin{aligned} \frac{dN}{dt}=\sum _{i=1}^n \frac{dN_i}{dt}\le&\sum _{i=1}^n \Lambda _i- \sum _{i=1}^n \mu _i N_i< {\bar{\Lambda }}- {\bar{\mu }} N, \end{aligned}$$

where ${\bar{\Lambda }}=\sum _{i=1}^n \Lambda _i$ and ${\bar{\mu }}=\min \{ \mu _i \}$. Therefore, $N(t)\le (N(0)-\frac{{\bar{\Lambda }}}{{\bar{\mu }}}) e^{-{\bar{\mu }}t}+\frac{{\bar{\Lambda }}}{{\bar{\mu }}}.$ This implies $N(t)\le \frac{{\bar{\Lambda }}}{{\bar{\mu }}}$ when $N(0)\le \frac{{\bar{\Lambda }}}{{\bar{\mu }}}$. Similarly, as in^37^, the first equation of system (1) is written as,

$$\begin{aligned} \frac{dS_i}{dt}\le \Lambda _i-\mu _i S_i+\sum _{\begin{array}{c} j=1,\\ j\ne i \end{array}}^{n}(a_{ij}S_j-a_{ji}S_i)=(b-G_0S)_i, \end{aligned}$$

where $$S=[S_i]_{n\times 1}$$, $$b=[\Lambda _i]_{n\times 1}$$ and

2 $$\begin{aligned} G_0= [\delta _{ij}(\mu _i+\sum _{\begin{array}{c} j=1,\\ j\ne i \end{array}}^{n}a_{ji})-(1-\delta _{ij})a_{ij}]_{n\times n}. \end{aligned}$$

and $\delta _{ij}$ denotes the Kronecker delta, defined as $\delta _{ij}=1$ for $i=j$ and $\delta _{ij}=0$ for $i\ne j$.

Hence, $\frac{dS_i}{dt}\le 0$ for $S_i= (G_0^{-1}b)_i=S_{i_0}$ (say) for each $i=1,2,\ldots ,n$. Since $G_0$ is a nonsingular ${\mathcal {M}}$- matrix, $G_0^{-1}$ is non negative and $(G_0^{-1}b)_i\ge 0$ (see^44^ for details) for all $i=1,2,\ldots ,n$. This completes the proof. $\square$

Here, this result emphasizes that all compartments representing the different stages of the disease in the population must not be negative and they should also remain bounded. A negative and unbounded population makes no sense in populations.

## Results

As mentioned earlier, the primary purpose of this study is to find out the impact of the movement of individuals in different patches. For the control of COVID-19 spread, various non-pharmaceutical interventions related to mobility, such as local confinement zones, no entry without negative tests, or even a complete travel ban, have been applied by healthcare personnel. Because of this, we shall consider our proposed model under three different cases for the *i*th patch- (i) when both immigration and emigration are allowed, (ii) when only emigration is allowed, and (iii) when there is complete isolation of the local patch from rest of the population. Here it is assumed that, in all three cases, the other patches are connected. In the following, we will analytically study these cases.

### Case 1: both immigration and emigration are allowed

In this section, we consider the case where both immigration and emigration are allowed in each patch *i* $(i=1,2,\ldots ,n$), which means the mobility parameters $a_{ij},a_{ji},b_{ij},b_{ji},c_{ij},c_{ji},d_{ij},d_{ji}\ne 0$ for $j\ne i$, $j=1,2,\ldots ,n$. This means that individuals can both move from patch *i* to any other patch and also other patch individuals can join the individuals in patch *i*. Under this assumption, we obtain the following results that hold for *i*th patch.

1. The unique disease-free equilibrium (DFE) for each patch *i* $(i=1,2,\ldots ,n)$ of the system (1) is $E_{0}^{(i)}=((G_0^{-1}b)_i,0,0,0,0,0,0)$ and the unique disease-free equilibrium of the multi-patch system is given as, $E_0^{(multi)}=((G_0^{-1}b)_1,0,0,0,0,0,0,(G_0^{-1}b)_2,$ $0,0,0,0,0,0, \ldots ,(G_0^{-1}b)_n,0,0,$ 0, 0, 0, 0). Here, $G_0$ is as defined in (2), $b=[\Lambda _i]_{n\times 1}$, and $(G_0^{-1}b)_i$ is *i* th element of $[G_0^{-1}b]_{n\times 1}$.
2. The basic reproduction number is given by
  3 $$\begin{aligned} {\mathscr {R}}_0^{(multi)}&= \rho (V_1V_{11}^{-1}V_{22}^{-1}+V_2 V_{11}^{-1}), \end{aligned}$$
  where $$V_{11}= [\delta _{ij}(\sigma _i+\mu _i+\sum _{\begin{array}{c} j=1,\\ j\ne i \end{array}}^{n}b_{ji})-(1-\delta _{ij})b_{ij}]_{n\times n}$$, $$V_{22}= [\delta _{ij}(\mu _i+\gamma _i+\theta _i+\sum _{\begin{array}{c} j=1,\\ j\ne i \end{array}}^{n}c_{ji})-(1-\delta _{ij})c_{ij}]_{n\times n}$$, $$V_1= \begin{bmatrix}\delta _{ij}(\sigma _iS_{i_0}\varepsilon _i\beta _{a_i}+\frac{\sigma _iS_{i_0}\beta _{s_i}}{\mu _{s_i}+\mu _i+\alpha _i+\alpha _{s_i}}(\frac{\varepsilon _i\gamma _i\eta _i}{\eta _i+\rho _i+\mu _i}+\varepsilon _i\theta _i))\end{bmatrix}_{n \times n}=[v_{1_{ij}}]_{n\times n}$$, and $$V_2= \begin{bmatrix}\delta _{ij} (\frac{\sigma _iS_{i_0}\beta _{s_i}(1-\varepsilon _i)}{\mu _{s_i}+\mu _i+\alpha _i+\alpha _{s_i}})\end{bmatrix}_{n\times n}=[v_{2_{ij}}]_{n\times n}$$, $$i=1,2,\ldots ,n$$, $$j=1,2,\ldots ,n$$.
3. The DFE $E_0^{(multi)}=(S_{1_0},0,0,0,0,0,0,S_{2_0},0,0,0,0,0,0,\ldots ,S_{n_0},0,0,0,0,0,0)$ is locally asymptotically stable for ${\mathscr {R}}_0^{(multi)}<1$ and unstable for ${\mathscr {R}}_0^{(multi)}>1$.
4. The DFE, $E_0^{(multi)}$ is globally asymptotically stable provided ${\mathscr {R}}_0^{(multi)}<1$ and $\nu _i=0$ for each $i=1,2,\ldots ,n$.
5. The endemic equilibrium $E_*^{(multi)}$ always exists for ${\mathscr {R}}_0^{(multi)}>1$.
6. The endemic equilibrium $E_{*}^{(multi)}=(S_{1_*},E_{1_*},I_{{a_1}_*},Q_{1_*},$ $I_{{s_1}_*},H_{1_*},R_{1_*},\ldots ,S_{n_*},E_{n_*},I_{{a_n}_*},Q_{n_*},I_{{s_n}_*},H_{n_*},R_{n_*})$ may exist for ${\mathscr {R}}_0^{(multi)}<1$. The condition of existence is that if
  4 $$\begin{aligned} (A_0+A_1D[I_{{s_i}_*}]+A_2D[I_{{s_i}_*}^2]+A_3 D[I_{{s_i}_*}^3]+A_4 D[I_{{s_i}_*}^4])I_{s_*}=0 \end{aligned}$$
  has a positive real root, where the coefficients are given in section “effect of mobility during epidemic” of the supplementary document.

### Case 2: only emigration is allowed

In this section, we explore the case when immigration of individuals is not allowed but emigration is allowed in patch *i* (for any fixed *i*,  $i=1,2,\ldots ,n$), i.e., $a_{ij}=b_{ij}=c_{ij}=d_{ij}=0$ and $a_{ji},b_{ji},c_{ji},d_{ji}\ne 0$ for $j\ne i$, $j=1,2,\ldots ,n$. Thus, in this case, only individuals from patch *i* can move out and go to the any other patch *j* but the individuals from other patches cannot enter the patch *i*. Under this assumption, we obtain the following results that hold for *i*th patch.

1. The DFE $${\tilde{E}}_0^{(i)}=({\tilde{S}}_{i_0},0,0,0,0,0,0)=(\frac{\Lambda _i}{\mu _i+\sum _{\begin{array}{c} j=1,\\ j\ne i \end{array}}^n a_{ji}},0,0,0,0,0,0)$$ exists always in $\Gamma$ for each *i* ($i=1,2,\ldots ,n$).
2. The basic reproduction number in patch *i* is given by
  5 $$\begin{aligned} \tilde{{\mathscr {R}}}_0^{(i)}=\frac{v_{1_{ii}}}{(\sigma _i+\mu _i+\sum _{\begin{array}{c} j=1,\\ j\ne i \end{array}}^n b_{ji})(\mu _i+\gamma _i+\theta _i+\sum _{\begin{array}{c} j=1,\\ j\ne i \end{array}}^n c_{ji})}+\frac{v_{2_{ii}}}{(\sigma _i+\mu _i+\sum _{\begin{array}{c} j=1,\\ j\ne i \end{array}}^n b_{ji})},\, i=1,2,\ldots ,n. \end{aligned}$$
  Again, when immigration of individuals is prohibited into patch *i* $(i=1,2,\ldots ,n)$, the infection caused by an individual during its symptomatic infectious period is given by
  $$\begin{aligned} \tilde{{\mathscr {R}}}_{0,s}^{(i)}=\frac{\beta _{s_i} {\tilde{S}}_{i_0}}{\mu _{s_i}+\mu _i+\alpha _{s_i}+\alpha _i}. \end{aligned}$$
  The infection caused by an individual initially asymptomatic is given by
  $$\begin{aligned} \tilde{{\mathscr {R}}}_{0,a}^{(i)}=\frac{\beta _{a_i} {\tilde{S}}_{i_0}}{\mu _i+\gamma _i+\theta _i+\sum _{\begin{array}{c} j=1,\\ j\ne i \end{array}}^n c_{ji}}+\left( \frac{\theta _i}{\mu _i+\gamma _i+\theta _i+\sum _{\begin{array}{c} j=1,\\ j\ne i \end{array}}^n c_{ji}}+\frac{\gamma _i}{\mu _i+\gamma _i+\theta _i+\sum _{\begin{array}{c} j=1,\\ j\ne i \end{array}}^n c_{ji}}\frac{\eta _i}{\mu _i+\rho _i+\eta _i}\right) \tilde{{\mathscr {R}}}_{0,s}^{(i)}, \end{aligned}$$
  where the last term reflects the route by which an individual initially asymptomatic can become symptomatic. Hence, $\bar{{\mathscr {R}}}_0^{(i)}$ in (5) can be expressed as
  $$\begin{aligned} \tilde{{\mathscr {R}}}_0^{(i)}=\frac{\sigma _i}{\sigma _i+\mu _i+\sum _{\begin{array}{c} j=1,\\ j\ne i \end{array}}^n b_{ji}}(\varepsilon \tilde{{\mathscr {R}}}_{0,a}^{(i)}+(1-\varepsilon ) \tilde{{\mathscr {R}}}_{0,s}^{(i)}), \end{aligned}$$
  where the factors $$\frac{\sigma _i}{\sigma _i+\mu _i+\sum _{\begin{array}{c} j=1,\\ j\ne i \end{array}}^n, b_{ji}}$$, for $i=1,2,\ldots ,n$, indicate the probability of not dying during the exposed period and so becoming infectious.
3. The DFE, ${\tilde{E}}_0^{(i)}$ is locally asymptotically stable if $\tilde{{\mathscr {R}}}_0^{(i)}<1$ and unstable if $\tilde{{\mathscr {R}}}_0^{(i)}>1$.
4. ${\tilde{E}}_0^{(i)}$ is globally asymptotically stable if $\tilde{{\mathscr {R}}}_0^{(i)}<1$ and $\nu _i=0$. Considering the Lyapunov function, $L_2= k_{1_i} E_i+ k_{2_i} I_{a_i}+k_{3_i} Q_i+k_{4_i} I_{s_i}$, we obtain the required conditions.
5. The endemic equilibrium is given by, ${\tilde{E}}_*^{(i)}=({\tilde{S}}_{i_*},{\tilde{E}}_{i_*},{\tilde{I}}_{{a_i}_*},{\tilde{Q}}_{i_*},{\tilde{I}}_{{s_i}_*},{\tilde{H}}_{i_*},{\tilde{R}}_{i_*})$ for each *i*, where the components are $${\tilde{E}}_{i_*}=\frac{\mu _i+\gamma _i+\theta _i+\sum _{\begin{array}{c} j=1,\\ j\ne i \end{array}}^n c_{ji}}{\varepsilon _i\sigma _i}{\tilde{I}}_{{a_i}_*}$$, ${\tilde{Q}}_{i_*}= \frac{\gamma _i{\tilde{I}}_{{a_i}_*}}{\eta _i+\rho _i+\mu _i}$, $${\tilde{I}}_{{a_i}_*}= \frac{(l_{i_1}+l_{i_2}{\tilde{I}}_{{s_i}_*})\varepsilon _i{\tilde{I}}_{{s_i}_*}}{(1+\nu _i {\tilde{I}}_{{s_i}_*})(\varepsilon _il_{i_3}+(1-\varepsilon _i)\sum _{\begin{array}{c} j=1,\\ j\ne i \end{array}}^n c_{ji})}$$, $${\tilde{S}}_{i_*}= \frac{(\sigma _i+\mu _i+\sum _{\begin{array}{c} j=1,\\ j\ne i \end{array}}^n b_{ji}){\tilde{E}}_i^*}{\frac{\beta _{s_i}}{1+m_{s_i} {\tilde{I}}_{s_i}^*}{\tilde{I}}_{{s_i}_*}+\frac{\beta _{a_i}}{1+m_{a_i}{\tilde{I}}_{{a_i}_*}}{\tilde{I}}_{{a_i}_*}}, \,\, {\tilde{H}}_{i_*}= \frac{\alpha _{s_i} {\tilde{I}}_{{s_i}_*}+\frac{\alpha _i {\tilde{I}}_{{s_i}_*}}{1+\nu _i {\tilde{I}}_{{s_i}_*}}}{\mu _{h_i}+\mu _i+\xi _i}$$, $${\tilde{R}}_{i_*}=\frac{\rho _i {\tilde{Q}}_{i_*}+\xi _i {\tilde{H}}_{i_*}}{\mu _i+\sum _{\begin{array}{c} j=1,\\ j\ne i \end{array}}^n d_{ji}}$$, and ${\tilde{I}}_{{s_i}_*}$ satisfies
  6 $$\begin{aligned} a_{i_4} {\tilde{I}}_{{s_i}_*}^4+ a_{i_3} {\tilde{I}}_{{s_i}_*}^3+a_{i_2} {\tilde{I}}_{{s_i}_*}^2+a_{i_1} {\tilde{I}}_{{s_i}_*}+a_{i_0}=0. \end{aligned}$$
  The coefficients in the above expression are,
  $$a_{i_4}= \frac{l_{i_2}^2l_{i_4}}{l_{i_3}}(\beta _{s_i}m_{s_i}+\beta _{a_i}m_{a_i}+(\mu _i+\sum _{\begin{array}{c} j=1,\\ j\ne i \end{array}}^n a_{ji})m_{s_i}m_{a_i}),$$
  $$a_{i_3}= \frac{l_{i_2}}{l_{i_3}}[(\mu _i+\sum _{\begin{array}{c} j=1,\\ j\ne i \end{array}}^n a_{ji})l_{i_4}\{m_{s_i}(\nu _il_{i_3}+m_{a_i}l_{i_1})+m_{a_i}(l_{i_2}+m_{s_i}l_{i_1})\}+l_{i_4}\{l_{i_1}(\beta _{s_i}m_{a_i}+$$
  $$\beta _{a_i}m_{s_i})+l_{i_5}\}- \Lambda _i\nu _il_{i_3}(\beta _{s_i}m_{a_i}+\beta _{a_i}m_{s_i})],$$
  $$a_{i_2}= \frac{1}{l_{i_3}}[(\mu _i+\sum _{\begin{array}{c} j=1,\\ j\ne i \end{array}}^n a_{ji})l_{i_4}\{m_{s_i}l_{i_2}l_{i_3}+(\nu _il_{i_3}+m_{a_i}l_{i_1})(l_{i_2}+m_{s_i}l_{i_1})+m_{a_i}l_{i_1}l_{i_2}\}+$$
  $$l_{i_4}\{l_{i_2}(\beta _{s_i}l_{i_3}+\beta _{a_i}l_{i_1})+ l_{i_1}l_{i_5}\}-\Lambda _il_{i_3}(\nu _il_{i_5}+l_{i_2}(\beta _{s_i}m_{a_i}+\beta _{a_i}m_{s_i})\}],$$
  $$a_{i_1}= l_{i_4}[(\mu _i+\sum _{\begin{array}{c} j=1,\\ j\ne i \end{array}}^n a_{ji})(l_{i_2}+m_{s_i}l_{i_1}+\nu _il_{i_1}+m_{a_i}\frac{l_{i_1}^2}{l_{i_3}})+\frac{l_{i_1}}{l_{i_3}}(\beta _{s_i}l_{i_3}+\beta _{a_i}l_{i_1})]-\Lambda _i(l_{i_5}+\nu _i(\beta _{s_i}l_{i_3}+\beta _{a_i}l_{i_1}))],$$
  $$a_{i_0}= (\mu _i+\sum _{\begin{array}{c} j=1,\\ j\ne i \end{array}}^n a_{ji})l_{i_1}l_{i_4}-\Lambda _i(\beta _{s_i}l_{i_3}+\beta _{a_i}l_{i_1})=(\mu _i+\sum _{\begin{array}{c} j=1,\\ j\ne i \end{array}}^n a_{ji})l_{i_1}l_{i_4}(1-\tilde{{\mathscr {R}}}_0^{(i)}),$$
  and
  $$l_{i_1}=\mu _{s_i}+\mu _i+\alpha _{s_i}+\alpha _i,$$
  $$l_{i_2}=\nu _i(\mu _{s_i}+\mu _i+\alpha _{s_i}),$$
  $$l_{i_3}=(\mu _i+\gamma _i+\theta _i)\frac{1-\varepsilon _i}{\varepsilon _i}+\frac{\eta _i\gamma _i}{\eta _i+\rho _i+\mu _i}+\theta _i,$$
  $${l_{i_4}=(\sigma _i+\mu _i+\sum _{\begin{array}{c} j=1,\\ j\ne i \end{array}}^n b_{ji})} \quad {\frac{\mu _i+\gamma _i+\theta _i+\sum _{\begin{array}{c} j=1,\\ j\ne i \end{array}}^n c_{ji}}{\varepsilon _i\sigma _i}}$$
  and
  $$l_{i_5}=\beta _{s_i}(\nu _iL_{i_3}+m_{a_i}l_{i_1})+\beta _{a_i}(m_{s_i}l_{i_1}+l_{i_2}).$$
  The number of possible positive real roots of Eq. (6) depends on coefficients $a_{i_4}$-$a_{i_0}$ as follows Table 3.
6. Endemic equilibrium, ${\tilde{E}}_*^{(i)}$ exists for $\tilde{{\mathscr {R}}}_0^{(i)}>1$ and may or may not exist for $\tilde{{\mathscr {R}}}_0^{(i)}<1$ for each *i*. As the endemic equilibrium ${\tilde{E}}_*^{(i)}$ exists if Eq. (6) has a real positive root, and the number of endemic equilibrium points will be equal to the number of real positive roots of (6). In Table 3 it is shown that a positive real ${\tilde{I}}_{{s_i}_*}$ exists for $\tilde{{\mathscr {R}}}_0>1$. So ${\tilde{E}}_*^{(i)}$ exists for $\tilde{{\mathscr {R}}}_0^{(i)}>1$. Again since real positive ${\tilde{I}}_{{s_i}_*}$ may or may not exist for $\tilde{{\mathscr {R}}}_0<1$ (by Table 3), so ${\tilde{E}}_*^{(i)}$ may or may not exist for $\tilde{{\mathscr {R}}}_0^{(i)}<1$.
7. Endemic equilibrium ${\tilde{E}}_*^{(i)}$ becomes locally asymptotically stable if
  7 $$\begin{aligned} \begin{aligned} {\left\{ \begin{array}{ll} &{} {\tilde{b}}_{i_1}{\tilde{b}}_{i_2}{\tilde{b}}_{i_3}{\tilde{b}}_{i_4}+ {\tilde{b}}_{i_0}{\tilde{b}}_{i_2}{\tilde{b}}_{i_3}+2{\tilde{b}}_{i_0}{\tilde{b}}_{i_1}{\tilde{b}}_{i_4}>{\tilde{b}}_{i_0}{\tilde{b}}_{i_3}^2{\tilde{b}}_{i_4}+{\tilde{b}}_{i_1}^2{\tilde{b}}_{i_4}^2 +{\tilde{b}}_{i_1}{\tilde{b}}_{i_2}^2+{\tilde{b}}_{i_0}^2,\\ &{}{\tilde{b}}_{i_2}{\tilde{b}}_{i_3}{\tilde{b}}_{i_4}+ {\tilde{b}}_{i_0}{\tilde{b}}_{i_4}>{\tilde{b}}_{i_2}^2+{\tilde{b}}_{i_1}{\tilde{b}}_{i_4}^2, {\tilde{b}}_{i_3}{\tilde{b}}_{i_4}>{\tilde{b}}_{i_2}, {\tilde{b}}_{i_4}>0, {\tilde{b}}_{i_0}>0. \end{array}\right. } \end{aligned} \end{aligned}$$
  holds, where
  $$\begin{aligned} {\tilde{b}}_{i_4}=&-(J_{i_{11}}+J_{i_{22}}+J_{i_{33}}+J_{i_{44}}+J_{i_{55}}),\\ {\tilde{b}}_{i_3}=&-J_{i_{23}} J_{i_{32}} + J_{i_{22}} J_{i_{33}} + J_{i_{22}} J_{i_{44}} + J_{i_{33}} J_{i_{44}} - J_{i_{25}} J_{i_{52}} + J_{i_{22}} J_{i_{55}} + J_{i_{33}} J_{i_{55}} +J_{i_{44}} J_{i_{55}} +J_{i_{11}}(J_{i_{22}} + J_{i_{33}} + J_{i_{44}} + J_{i_{55}}),\\ {\tilde{b}}_{i_2}=&-J_{i_{13}} J_{i_{21}} J_{i_{32}} + J_{i_{23}} J_{i_{32}} J_{i_{44}} - J_{i_{22}} J_{i_{33}} J_{i_{44}} - J_{i_{15}} J_{i_{21}} J_{i_{52}} + J_{i_{25}} J_{i_{33}} J_{i_{52}} + J_{i_{25}} J_{i_{44}} J_{i_{52}}- J_{i_{25}}J_{i_{32}} J_{i_{53}} + J_{i_{23}} J_{i_{32}} J_{i_{55}} - J_{i_{22}}\\&J_{i_{33}} J_{i_{55}} - J_{i_{22}} J_{i_{44}} J_{i_{55}} - J_{i_{33}} J_{i_{44}} J_{i_{55}} + J_{i_{11}} (J_{i_{23}} J_{i_{32}} - J_{i_{33}}J_{i_{44}} +J_{i_{25}} J_{i_{52}} - J_{i_{33}} J_{i_{55}} - J_{i_{44}} J_{i_{55}} - J_{i_{22}} (J_{i_{33}} + J_{i_{44}} + J_{i_{55}})), \\ {\tilde{b}}_{i_1}=&J_{i_{15}} J_{i_{21}} J_{i_{33}} J_{i_{52}} + J_{i_{15}} J_{i_{21}} J_{i_{44}} J_{i_{52}} - J_{i_{25}} J_{i_{33}} J_{i_{44}} J_{i_{52}} - J_{i_{15}} J_{i_{21}} J_{i_{32}} J_{i_{53}} + J_{i_{25}} J_{i_{32}} J_{i_{44}} J_{i_{53}} - J_{i_{25}}J_{i_{32}} J_{i_{43}} J_{i_{54}} -J_{i_{23}} J_{i_{32}} J_{i_{44}}\\&J_{i_{55}} + J_{i_{22}} J_{i_{33}} J_{i_{44}} J_{i_{55}} + J_{i_{13}} J_{i_{21}} J_{i_{32}} (J_{i_{44}} + J_{i_{55}}) + J_{i_{11}}(-J_{i_{25}} J_{i_{33}} J_{i_{52}} -J_{i_{25}} J_{i_{44}} J_{i_{52}} + J_{i_{25}} J_{i_{32}} J_{i_{53}} + J_{i_{33}} J_{i_{44}} J_{i_{55}} -J_{i_{23}}\\&J_{i_{32}} (J_{i_{44}} +J_{i_{55}}) + J_{i_{22}} (J_{i_{44}} J_{i_{55}} + J_{i_{33}} (J_{i_{44}} +J_{i_{55}}))),\\ {\tilde{b}}_{i_0}=&-J_{i_{15}} J_{i_{21}} (J_{i_{33}} J_{i_{44}} J_{i_{52}} - J_{i_{32}} J_{i_{44}} J_{i_{53}} + J_{i_{32}} J_{i_{43}} J_{i_{54}}) - J_{i_{13}} J_{i_{21}} J_{i_{32}} J_{i_{44}} J_{i_{55}} +J_{i_{11}} (J_{i_{25}} J_{i_{33}} J_{i_{44}}J_{i_{52}} - J_{i_{25}} J_{i_{32}} J_{i_{44}} J_{i_{53}} +\\&J_{i_{25}} J_{i_{32}} J_{i_{43}} J_{i_{54}} +J_{i_{23}} J_{i_{32}} J_{i_{44}} J_{i_{55}} - J_{i_{22}} J_{i_{33}} J_{i_{44}} J_{i_{55}}), \end{aligned}$$
  and $$J_{i_{11}}=-(\mu _i+\frac{\beta _{s_i}{\tilde{I}}_{{s_i}_*}}{1+m_{s_i} {\tilde{I}}_{{s_i}_*}}+\frac{\beta _{a_i}{\tilde{I}}_{{a_i}_*}}{1+m_{a_i}{\tilde{I}}_{{a_i}_*}}+\sum _{\begin{array}{c} j=1,\\ j\ne i \end{array}}^n a_{ji})=-\frac{\Lambda _i}{{\tilde{S}}_{i_*}}$$, $$J_{i_{13}}= -\frac{\beta _{a_i}{\tilde{S}}_{i_*}}{(1+m_{a_i}{\tilde{I}}_{{a_i}_*})^2}$$, $$J_{i_{15}}= -\frac{\beta _{s_i}{\tilde{S}}_{i_*}}{(1+m_{s_i}{\tilde{I}}_{{s_i}_*})^2}$$, $$J_{i_{21}}= \frac{\beta _{s_i}{\tilde{I}}_{{s_i}_*}}{1+m_{s_i} {\tilde{I}}_{{s_i}_*}}+\frac{\beta _{a_i}{\tilde{I}}_{{a_i}_*}}{1+m_{a_i}{\tilde{I}}_{{a_i}_*}}$$, $$J_{i_{22}}= -(\sigma _i+\mu _i+\sum _{\begin{array}{c} j=1,\\ j\ne i \end{array}}^n b_{ji})$$, $$J_{i_{23}}= \frac{\beta _{a_i}{\tilde{S}}_{i_*}}{(}{1+m_{a_i}{\tilde{I}}_{{s_i}_*}}$$, $$J_{i_{25}}=\frac{\beta _{s_i}{\tilde{S}}_{i_*}}{(1+m_{s_i}{\tilde{I}}_{{s_i}_*})^2}$$, $$J_{i_{32}}= \varepsilon _i\sigma _i$$, $$J_{i_{33}}=-(\mu _i+\gamma _i+\theta _i+\sum _{\begin{array}{c} j=1,\\ j\ne i \end{array}}^n c_{ji})$$, $$J_{i_{43}}= \gamma _i$$, $$J_{i_{44}}=-(\eta _i+\rho _i+\mu _i)$$, $$J_{i_{52}}=(1-\varepsilon _i)\sigma _i$$, $$J_{i_{53}}=\theta _i$$, $$J_{i_{54}}=\eta _i$$, $$J_{i_{55}}=-(\mu _{s_i}+\mu _i+\alpha _{s_i}+\frac{\alpha _i}{(1+\nu _i {\tilde{I}}_{{s_i}_*})^2})$$.

**Table 3:** Number of possible positive real roots of Eq. (6).

Cases	Coefficients					Number of possible
	$a_{i_4}$	$a_{i_3}$	$a_{i_2}$	$a_{i_1}$	$a_{i_0}$	Positive roots
$\tilde{{\mathscr {R}}}_0^{(i)}<1$	+	+/−	+/−	+/−	+	4/2/0
$\tilde{{\mathscr {R}}}_0^{(i)}>1$	+	+/−	+/−	+/−	–	3/1

#### Theorem 3.1

Suppose, for a fixed *i*, $a_{ij}=b_{ij}=c_{ij}=d_{ij}=0$ for $j\ne i$, $j=1,2,\ldots ,n$ and $\tilde{{\mathscr {R}}}_0^{(i)}=1$

$$\beta _a=\beta _{a_i}^*=\frac{(\sigma _i+\mu _i+\sum _{\begin{array}{c} j=1,\\ j\ne i \end{array}}^n b_{ji})(\mu _i+\gamma _i+\theta _i+\sum _{\begin{array}{c} j=1,\\ j\ne i \end{array}}^n c_{ji})-(\frac{\sigma _iS_{i_0}\beta _{s_i}}{\mu _{s_i}+\mu _i+\alpha _i+\alpha _{s_i}}(\frac{\varepsilon _i\gamma _i\eta _i}{\eta _i+\rho _i+\mu _i}+\varepsilon _i\theta _i))+v_{2_{ii}}(\mu _i+\gamma _i+\theta _i+\sum _{\begin{array}{c} j=1,\\ j\ne i \end{array}}^n c_{ji})}{\sigma _iS_{i_0}\varepsilon _i}$$

i.e., Then system (1) undergoes forward (transcritical) bifurcation if $\nu <\nu _i^*$ and a backward bifurcation if $\nu >\nu _i^*$ where,

$$\nu _i^*=$$ $$\frac{\frac{l_{i_1}\varepsilon _i^2\sigma _i^2(m_{a_i}\beta _{s_i}+m_{s_i}\beta _{a_i}^*)\beta _{a_i}^*S_{i_0}^2}{(\mu _i+\gamma _i+\theta _i+\sum _{\begin{array}{c} j=1,\\ j\ne i \end{array}}^{n}c_{ji})^2}-l_{i_1}\left\{ \frac{2\varepsilon _i\sigma _im_{s_i}\beta _{a_i}^*S_{i_0}}{\mu _i+\gamma _i+\theta _i+\sum _{\begin{array}{c} j=1,\\ j\ne i \end{array}}^{n}c_{ji}}+\beta _{s_i}\right\} (\sigma _i+\mu _i+\sum _{\begin{array}{c} j=1,\\ j\ne i \end{array}}^n b_{ji})+(\sigma _i+\mu _i+\sum _{\begin{array}{c} j=1,\\ j\ne i \end{array}}^n b_{ji})^2m_{s_i}l_{i_1}}{\alpha _i(\sigma _i+\mu _i+\sum _{\begin{array}{c} j=1,\\ j\ne i \end{array}}^n b_{ji}-\beta _{a_i}^*S_{i_0}\frac{\varepsilon _i\sigma _i}{\mu _i+\gamma _i+\theta _i+\sum _{\begin{array}{c} j=1,\\ j\ne i \end{array}}^{n}c_{ji}})^2}$$.

#### Remark 3.1

One of the necessary conditions for non existence of ${\tilde{E}}_*^{(i)}$ for $\tilde{{\mathscr {R}}}_0^{(i)}<1$ is $\nu _i=0$. Because the DFE ${\tilde{E}}_0^{(i)}$ is global asymptotically stable here and so disease will extinct for $\tilde{{\mathscr {R}}}_0^{(i)}<1$ under the condition $\nu _i=0$. Besides since the endemic equilibrium ${\tilde{E}}_*^{(i)}$ always exists for $\tilde{{\mathscr {R}}}_0^{(i)}>1$, so disease will persist for $\tilde{{\mathscr {R}}}_0^{(i)}>1$. This behaviour of the disease system is found due to taking saturated hospital facilities in the system (for details, see^41^).

### Case 3: neither immigration nor emigration is allowed

In this section, we consider the case where both immigration and emigration are not allowed in patch *i*, for any fixed *i*,  $i=1,2,\ldots ,n$. Hence, $a_{ij}=a_{ji}=b_{ij}=b_{ji}=c_{ij}=c_{ji}=d_{ij}=d_{ji}=0$ for $j\ne i$, and $j=1,2,\ldots ,n$. Then the fixed *i*th patch is separated from the rest of the multipatch system and acts as a single patch. Thus, in this case, no inter-patch movement is allowed and hence all the patches are isolated. In this case, we obtain the following results for the fixed *i*th patch.

1. The DFE ${\bar{E}}_0^{(i)}=({\bar{S}}_{i_0},0,0,0,0,0,0)=(\frac{\Lambda _i}{\mu _i},0,0,0,0,0,0)$ exists always in $\Gamma$.
2. The basic reproduction number in patch *i* is given by
  8 $$\begin{aligned} \bar{{\mathscr {R}}}_0^{(i)}=\frac{v_{1_{ii}}}{(\sigma _i+\mu _i)(\mu _i+\gamma _i+\theta _i)}+\frac{v_{2_{ii}}}{(\sigma _i+\mu _i)},\, i=1,2,\ldots ,n. \end{aligned}$$
  Similarly as the above case, $\bar{{\mathscr {R}}}_0^{(i)}$ in (8) can be expressed as $\bar{{\mathscr {R}}}_0^{(i)}=\frac{\sigma }{\sigma +\mu }(\varepsilon \bar{{\mathscr {R}}}_{0,a}^{(i)}+(1-\varepsilon ) \bar{{\mathscr {R}}}_{0,s}^{(i)}).$
3. The DFE, ${\bar{E}}_0^{(i)}$ is locally asymptotically stable if $\bar{{\mathscr {R}}}_0^{(i)}<1$, globally asymptotically stable if $\bar{{\mathscr {R}}}_0^{(i)}<1$ and $\nu _i=0$ and unstable if $\bar{{\mathscr {R}}}_0^{(i)}>1$.
4. The endemic equilibrium is given by ${\bar{E}}_*^{(i)}=({\bar{S}}_{i_*},{\bar{E}}_{i_*},{\bar{I}}_{{a_i}_*},{\bar{Q}}_{i_*},{\bar{I}}_{{s_i}_*},{\bar{H}}_{i_*},{\bar{R}}_{i_*})$, where $({\bar{S}}_{i_*},{\bar{E}}_{i_*},{\bar{I}}_{{a_i}_*},$ ${\bar{Q}}_{i_*},{\bar{I}}_{{s_i}_*},{\bar{H}}_{i_*}$ $,{\bar{R}}_i^*)=({\tilde{S}}_{i_*},{\tilde{E}}_{i_*},{\tilde{I}}_{{a_i}_*},{\tilde{Q}}_{i_*},{\tilde{I}}_{{s_i}_*},{\tilde{H}}_{i_*},{\tilde{R}}_{i_*})|_{a_{ji}=b_{ji}=c_{ji}=d_{ji}=0}$.
5. Endemic equilibrium, ${\bar{E}}_*^{(i)}$ exists for $\bar{{\mathscr {R}}}_0^{(i)}>1$ and may or may not exist for $\bar{{\mathscr {R}}}_0^{(i)}<1$.
6. Endemic equilibrium ${\bar{E}}_*^{(i)}$ becomes locally asymptotically stable if
  9 $$\begin{aligned} \begin{aligned} {\left\{ \begin{array}{ll} &{} {\bar{b}}_{i_1}{\bar{b}}_{i_2}{\bar{b}}_{i_3}{\bar{b}}_{i_4}+{\bar{b}}_{i_0}{\bar{b}}_{i_2}{\bar{b}}_{i_3}+2{\bar{b}}_{i_0}{\bar{b}}_{i_1}{\bar{b}}_{i_4}>{\bar{b}}_{i_0}{\bar{b}}_{i_3}^2{\bar{b}}_{i_4}+{\bar{b}}_{i_1}^2{\bar{b}}_{i_4}^2 +{\bar{b}}_{i_1}{\bar{b}}_{i_2}^2+{\bar{b}}_{i_0}^2,\\ &{}{\bar{b}}_{i_2}{\bar{b}}_{i_3}{\bar{b}}_{i_4}+{\bar{b}}_{i_0}{\bar{b}}_{i_4}>{\bar{b}}_{i_2}^2+{\bar{b}}_{i_1}{\bar{b}}_{i_4}^2,{\bar{b}}_{i_3}{\bar{b}}_{i_4}>{\bar{b}}_{i_2},{\bar{b}}_{i_4}>0,{\bar{b}}_{i_0}>0. \end{array}\right. } \end{aligned} \end{aligned}$$
  holds, where $$({\bar{b}}_{i_4},{\bar{b}}_{i_3},{\bar{b}}_{i_2},{\bar{b}}_{i_1},{\bar{b}}_{i_0})=({\tilde{b}}_{i_4},{\tilde{b}}_{i_3},{\tilde{b}}_{i_2},{\tilde{b}}_{i_1},{\tilde{b}}_{i_0})|_{a_{ji}=b_{ji}=c_{ji}=d_{ji}=0}$$.

#### Remark 3.2

$$\tilde{{\mathscr {R}}}_0^{(i)} in~ \,(5)<\bar{{\mathscr {R}}}_0^{(i)}$$ in (8).

#### Theorem 3.2

The basic reproduction number in each patch is bounded by the basic reproduction number of the whole multi-patch systems, i.e., $\max _{1\le i\le n}\tilde{{\mathscr {R}}}_0^{(i)}\le {\mathscr {R}}_0^{(multi)}$. Further, if rates $\varepsilon _i$, $\sigma _i$, $\eta _i$, $\rho _i$, $\mu _i$, $\gamma _i$, $\alpha _i$ are fixed in each patch, then

$$\begin{aligned} \min _{1\le i\le n}\bar{{\mathscr {R}}}_0^{(i)}\le {\mathscr {R}}_0^{(multi)}\le \max _{1\le i\le n}\bar{{\mathscr {R}}}_0^{(i)}. \end{aligned}$$

#### Proof

We denote $Y_1=[y_{1_{ij}}]_{n\times n}=V_{11}^{-1}\ge 0$, $Y_2=[y_{2_{ij}}]_{n\times n}=V_{22}^{-1}\ge 0$ (as $V_{11}$, $V_{22}$ are ${\mathscr {M}}$- matrix^44^) and $V_1V_{11}^{-1}V_{22}^{-1}+V_2 V_{11}^{-1}=W=[w_{ij}]_{n\times n}$ such that

10 $$\begin{aligned} w_{ij}= v_{1_{ii}}\sum _{{k}=1}^n y_{1_{ik}}y_{2_{kj}}+v_{2_{ii}} y_{1_{ij}}. \end{aligned}$$

By Fischer’s inequality^45^ we get,

11 $$\begin{aligned}&\det {V_{11}}\le (\sigma _i+\mu _i+\sum _{\begin{array}{c} j=1,\\ j\ne i \end{array}}^{n}b_{ji})\det {V_{11}[i']}, \quad \det {V_{22}}\le (\mu _i+\gamma _i+\theta _i+\sum _{\begin{array}{c} j=1,\\ j\ne i \end{array}}^{n}c_{ji})\det {V_{22}[i']}. \end{aligned}$$

where $V_{11}[i']$ and $V_{22}[i']$ denote the sub-matrices of order $(n-1)\times (n-1)$ obtained by removing the *i*th row and the *i*th column of $V_{11}$ and $V_{22}$, respectively.

Now, using (5) and (11) in (10) we obtain,

$$\begin{aligned} w_{ii}=&v_{1_{ii}}\sum _{{k}=1}^n y_{1_{i{k}}}y_{2_{{k}i}}+v_{2_{ii}} y_{1_{i{i}}}\ge (v_{1_{ii}}y_{2_{ii}}+v_{2_{ii}}) y_{1_{ii}} = (v_{1_{ii}}\frac{\det {V_{22}[i']}}{\det {V_{22}}}+v_{2_{ii}})\frac{\det {V_{11}[i']}}{\det {V_{11}}}\ge \tilde{{\mathscr {R}}}_0^{(i)}. \end{aligned}$$

By Corollary 8.1.20^46^, it follows that ${\mathscr {R}}_0^{(multi)}=\rho (W)\ge w_{ii}\ge \tilde{{\mathscr {R}}}_0^{(i)}$, for $i=1,2,\ldots ,n$. Hence, we get $\max _{1\le i\le n}\tilde{{\mathscr {R}}}_0^{(i)}\le {\mathscr {R}}_0^{(multi)}.$

Consider that for each $i=1,2,\ldots ,n$, $\varepsilon _i=\varepsilon$, $\sigma _i=\sigma$, $\eta _i=\eta$, $\rho _i=\rho$, $\mu _i=\mu$, $\mu _{s_i}=\mu _{s}$, $\mu _{a_i}=\mu _{a}$, $\gamma _i=\gamma$, $\theta _i=\theta$, $\alpha _{s_i}=\alpha _s$, $\alpha _i=\alpha$ are constant, but the transaction rates $\beta _{s_i}$ and $\beta _{a_i}$ may be different in each patch. Without loss of generality, we assume $\beta _{s_1}\le \beta _{s_2}\le \ldots \le \beta _{s_n}$ and $\beta _{a_1}\le \beta _{a_2}\le \ldots \le \beta _{a_n}$. Since sum of each column of matrix $V_{11}$, $V_{22}$ are $\sigma _i+\mu _i=\sigma +\mu$, $\mu _i+\gamma _i+\theta _i=\mu +\gamma +\theta$ respectively, so $\sum _{i=1}^n y_{1_{ij}}= \frac{1}{\sigma +\mu }$ and $\sum _{i=1}^n y_{2_{ij}}= \frac{1}{\mu +\gamma +\theta }$ for $j=1,2,\ldots ,n$. Then using (8) we have,

$$\begin{aligned} \sum _{i=1}^n w_{ij}=&\sum _{i=1}^n (v_{1_{ii}}\sum _{{k}=1}^n y_{1_{i{k}}}y_{2_{{k}j}}+v_{2_{ii}} y_{1_{i{j}}}),\\ \le&\left( \sigma S_{i_0}\varepsilon \beta _{a_n}+\frac{\sigma S_{i_0}\beta _{s_n}}{\mu _{s}+\mu +\alpha +\alpha _{s}}(\frac{\varepsilon \gamma \eta }{\eta +\rho +\mu }+\varepsilon \theta )\right) \sum _{{k}=1}^n (\sum _{i=1}^ny_{1_{ik}})y_{2_{kj}}+\frac{\sigma S_{i_0}\beta _{s_n}(1-\varepsilon )}{\mu _{s}+\mu +\alpha +\alpha _{s}}(\sum _{i=1}^ny_{1_{ik}}),\\ =&\frac{v_{1_{nn}}}{(\sigma +\mu )(\mu +\gamma +\theta )}+\frac{v_{2_{nn}}}{(\sigma +\mu )}= \bar{{\mathscr {R}}}_0^{(n)}. \end{aligned}$$

Similarly we have $\sum _{i=1}^n w_{ij}\ge \frac{v_{1_{11}}}{(\sigma +\mu )(\mu +\gamma +\theta )}+\frac{v_{2_{11}}}{(\sigma +\mu )}= \bar{{\mathscr {R}}}_0^{(1)}$. Since, $\rho (W)$ lies between the minimum and maximum column sums of *W*^46^, therefore, $\min _{1\le i\le n}\bar{{\mathscr {R}}}_0^{(i)}\le {\mathscr {R}}_0^{(multi)}=\rho (W)< \max _{1\le i\le n}\bar{{\mathscr {R}}}_0^{(i)}$. $\square$

## Parameter estimation and short time prediction

For the purpose of parameter estimation, we have used data of COVID-19 active cases from 3 Indian states, namely, Kerala, Maharashtra, and Tamil Nadu, for the period of 14 July to 20 August 2021^47^. During the study time, they were the most affected neighbouring states in India with considerable connectivity. So we consider $n=3$ in the proposed model (1), where we represent Kerala as patch 1, Maharashtra as patch 2, and Tamil Nadu as patch 3. As per report^47^, in India, including Maharastra and Tamil Nadu total per day active cases is decreasing, but in Kerala, the total number of active cases per day is increasing. During the study, nearly 50% of total COVID-19 cases in India are from Kerala.

We have assumed that all COVID-19 active cases are either hospitalized or isolated. Therefore, we use the COVID-19 active cases into the *H* compartment of our model. We assume and fix some parametric values as shown in Table 2 and estimate parameters $\beta _{s_i}$, $\gamma _i$ and $\alpha _{s_i}$ from the above-mentioned data. Also, we assume that mobility rates ($p_{12}=p_{21}=p_{23}=p_{32}=p_{13}=p_{31}=0.001$) are equal for all compartments in each patch so that our mobility matrices are- $A=B=C=D=P=[p_{ij}]_{3 \times 3}$ (say) for $i\ne j$,$i,j=1,2,3$ with $p_{ii}=0$ for $i=1,2,3$.

**Figure 2:**
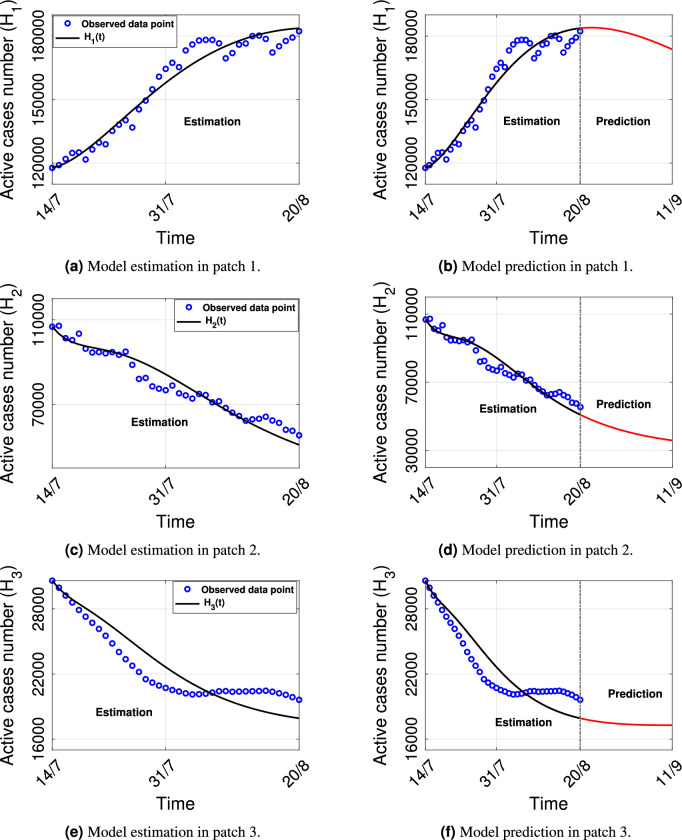
Plot of model fitting curve and observed data point of active COVID-19 cases in (**a**)–(**b**) patch 1 (Kerala), (**c**)–(**d**) patch 2 (Maharashtra), (**e**)–(**f**) patch 3 (Tamil Nadu). Black curve: model estimation from 14th July to 20th August; Red curve: model prediction from 21th August to 11th September; Blue circle: observed data from 14th July to 20th August.

The MATLAB software^48^ is used to fit the simulated and observed COVID-19 active cases for all these three states’ data during the mentioned time period using the *least square method* and MATLAB’s built-in function *lsqcurvefit*. We estimate the parameters $\beta _{s_i}$, the effective contact rate between susceptible and symptomatic individuals, $\gamma _{i}$, the quarantine rate of asymptomatic individuals, and $\alpha _{s_i}$, the quarantine rate of symptomatic individuals for $i=1,2,3$. The estimated values are shown in Table 4. The model fitting is depicted in Fig. 2a,c,e with actual COVID-19 active cases for all three data sets. In these figures, blue dots represent the observed COVID-19 active cases data, and the solid black curve represents the corresponding fitted curve for $H_i (i=1,2,3)$ population from the proposed model system.

**Table 4:** Estimated values of parameters $\beta _{s_i}$, $\gamma _i$, $\alpha _{s_i}$ for $i=1,2,3$ in system (1).

States	Estimated parametric values
Kerala (patch 1)	$\beta _{s_1}=0.0002$, $\gamma _1=0.15$, $\alpha _{s_1}=0.09$
Maharashtra (patch 2)	$\beta _{s_2}=0.00016$, $\gamma _2=0.15$, $\alpha _{s_2}=0.13$
Tamil Nadu (patch 3)	$\beta _{s_3}=0.0001$, $\gamma _3=0.16$, $\alpha _{s_3}=0.22$

After parameter estimation, we are interested in short-term predictions for each state, so using the parameters from Table 2 and estimated parameters from Table 4 we have predicted the number of new active COVID-19 cases in each patch for the next 22 days, i.e., from 21st August to 11th September 2021. These are shown in corresponding Fig. 2b,d,f, respectively, for Kerala, Maharashtra and Tamil Nadu. It is noticeable that eventually, the number of cases will reduce in each patch with time.

### Effect of mobility during epidemic

**Figure 3:**
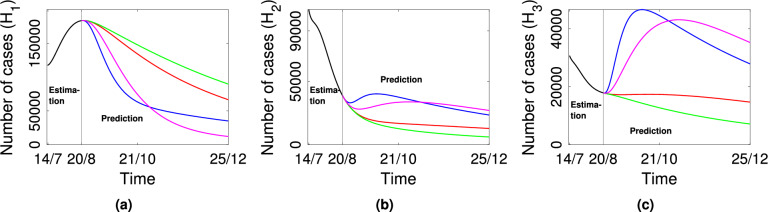
Plot of predicted active cases in all three patches for different mobility rates. Black curve: estimation curve, Red curve: prediction curve when all patches are connected with the same mobility rate i.e., $p_{12}=p_{21}=p_{23}=p_{32}=p_{13}=p_{31}=$ 0.001; Blue curve: prediction curve when mobility rates within all patches are increased i.e., $p_{21}=p_{31}=p_{12}=p_{13}=p_{23}$ $=p_{32}=0.05$; Green curve: prediction curve when immigration rate in patch 1 is higher than emigration rate i.e., $p_{12}=p_{13}$ $=0.01$, $p_{21}=p_{31}=p_{23}=p_{32}=0.001$; Magenta curve: prediction curve when emigration rate from patch 1 is higher than immigration rate i.e., $p_{21}=p_{31}=0.01$, $p_{12}=p_{13}=p_{23}=p_{32}=0.001$.

As we have already highlighted, one of our main objectives in this work is to understand the effect of mobility on disease spread. Hence, we numerically simulate the model system for above mentioned 3 patch set-ups. First, we numerically calculate the number of active cases in each patch for about four months, considering the estimated values. We choose parametric values as in Tables 2 and 4 with equal baseline mobility rates, i.e., $p_{12}=p_{21}=p_{23}=p_{32}=p_{13}=p_{31}=0.001$. The number of active cases, in this case, is represented by the red curve in Fig. 3. After that, we compare the active case number in each patch when mobility rates are varied. In each case, we get ${\mathscr {R}}_0^{(multi)}>1$, so the disease will survive in the system in all the following cases.

- If mobility rates are increased between patches i.e., $p_{12}=p_{21}=p_{13}=p_{31}=p_{23}=p_{32}=0.05$ (gives ${\mathscr {R}}_0^{(multi)}=1.96>1$), then the number of active cases will decrease in patch 1 and will increase in patch 2 and patch 3. The blue curve represents this outcome in Fig. 3 for all three patches.
- If immigration rate in patch 1 is higher than emigration rate i.e., $p_{12}=p_{13}=0.01$, $p_{21}=p_{31}=p_{23}=p_{32}=0.001$ (gives ${\mathscr {R}}_0^{(multi)}=1.55>1$), the number of active cases will increase in patch 1 and will decrease in patch 2 and patch 3. The green curve represents this outcome in Fig. 3.
- If emigration rate from patch 1 is higher than immigration rate i.e., $p_{21}=p_{31}=0.01$, $p_{12}=p_{13}=p_{23}=p_{32}=0.001$ (gives ${\mathscr {R}}_0^{(multi)}=3.6>1$), then the number of active cases will decrease in patch 1 and will increase in patch 2 and patch 3. The magenta curve represents this outcome in Fig. 3.

In the above, we have explained the scenario where all patches are connected directly via mobility, and the rates of mobility are varied. We note from the outcome that increased mobility in all patches reduces active cases in patch 1 but increases cases in the other two states. Further, increased emigration in Kerala minimizes the burden on Kerala but causes a wave in Tamil Nadu. Similarly, if we reduce immigration from Kerala, it significantly reduces the load on both Maharashtra and Tamil Nadu. These observations are interesting as, by restricting the mobility of the high load state, we can significantly control the spread of the disease.

**Figure 4:**
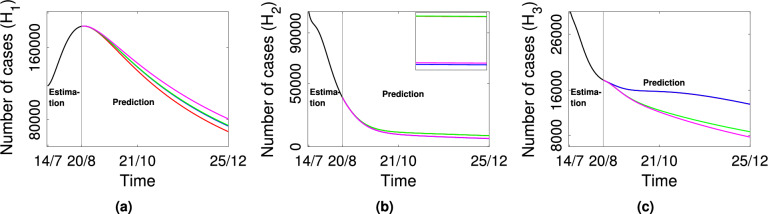
Plot of predicted active cases in all three patches for different mobility rates. Black curve: estimation curve, Red curve: prediction curve when all patches are connected with same mobility rate i.e., $p_{12}=p_{21}=p_{23}=p_{32}=p_{13}=p_{31}=$ 0.001; Blue curve: prediction curve when no mobility between patch 1 and patch 2 i.e., $p_{12}=p_{21}=0$, $p_{23}=p_{32}=p_{13}=$ $p_{31}=0.001$; Green curve: prediction curve when no mobility between patch 1 and patch 3 i.e., $p_{12}=p_{21}=p_{23}=p_{32}=0.001$, $p_{13}=p_{31}=0$; Magenta curve: prediction curve when all patches are isolated i.e., $p_{12}=p_{21}=p_{13}=p_{31}=p_{23}=p_{32}=0$.

Now we study the system behaviour when mobility is restricted between any two patches or among all the patches. For this, we assume $\beta _{s_2}=0.00006$ and $\beta _{s_3}=0.00008$ and remaining all parameters are fixed as mentioned in Tables 2 and 4 and mobility rates are same $p_{12}=p_{21}=p_{23}=p_{32}=p_{13}=p_{31}=0.001$ in all the patches. Then we obtain ${\mathscr {R}}_0^{(multi)}=2.539>1$. Therefore, the disease will persist in all three patches. The red curve represents the active case number in each patch in Fig. 4. Now, we consider the following cases to establish the mobility impact on disease persistence and extinction.

- If mobility between patch 1 and patch 2 is only restricted i.e., $p_{12}=p_{21}=0$, $p_{23}=p_{32}=p_{13}=p_{31}=0.001$ (implies ${\mathscr {R}}_0^{(multi)}=2.558$), then the number of active cases will increase in patch 1 and patch 3 and will decrease in patch 2 only. The blue curve represents this result in Fig. 4.
- If mobility between patch 1 and patch 3 is only restricted i.e., $p_{12}=p_{21}=p_{23}=p_{32}=0.001$, $p_{13}=p_{31}=0$ (implies ${\mathscr {R}}_0^{(multi)}=2.558$), then the number of active cases will increase in patch 1 and patch 2 and will decrease in patch 3. The green curve exhibits this outcome in Fig. 4.
- If mobility is restricted between all the patches i.e., $p_{12}=p_{21}=p_{23}=p_{32}=p_{13}=p_{31}=0$ (implies $\bar{{\mathscr {R}}}_0^{(1)}=2.57$, $\bar{{\mathscr {R}}}_0^{(2)}=0.89$, $\bar{{\mathscr {R}}}_0^{(3)}=0.86$ evaluated using (8)), then the number of active cases will increase in patch 1 and will decrease in patch 2 and patch 3. The magenta curve represents this outcome in Fig. 4.

Hence, we conclude if mobility is restricted among all patches, the disease load will be reduced in all the patches. Further, if mobility is restricted from a higher prevalence state (Kerala), then the disease load in that state will be low. Hence, we conclude that travel restrictions from high-prevalence states may reduce or control disease spread in other states.

## Discussion

In this paper, we propose and analyse a multi-patch model of COVID-19 disease transmission where each patch consists of seven compartments. Patches are connected through the inter-patch movement of individuals in susceptible, exposed, asymptomatic, and recovered compartments. We also accounted for the inhibitory effects of healthy individuals on incidence rate and the limitation of medical resources in the model system. We studied the multi-patch system having two or more patches. One of the important factors considered in our work is saturated incidence rates which are saturated by $\frac{\beta _{s_i}}{m_{s_i}}$ and $\frac{\beta _{a_i}}{m_{a_i}}$ where $\beta _{s_i}$, $\beta _{a_i}$ represent the effective contact rates between susceptible and infected and $m_{s_i}$, $m_{a_i}$ represent the early precaution measures. To the best of our knowledge, no multi-patch model of COVID-19 disease is analysed by taking a saturated incidence rate. The basic reproduction number of the multi-patch system is calculated in Eq. (3). We establish the existence of disease-free equilibrium and endemic equilibrium. One of the main goals of this work is to capture the mobility effect in disease spreading during the epidemic period. Therefore we study three cases-(i) when both immigration and emigration are allowed, (section “case 1: both immigration and emigration are allowed”), (ii) when only emigration is allowed (section “case 2: only emigration is allowed”) and (iii) when there is complete isolation of the local patch from rest of the population (section “case 3: neither immigration nor emigration is allowed”). It is observed that basic reproduction number of case 2 is less than case 1 (Theorem 3.2) and case 3 (Remark 3.2), which signifies that the basic reproduction number decreases as mobility rates increase.

The multi-patch model is designed numerically into a three-patch model where the data fitting is provided with the help of an example of three states in India, taken as the three patches. Our objective is to study the COVID-19 disease outbreak in these three states. As per reported cases in MoHFW, Kerala is the highest prevalence state in India of COVID-19 disease at the end of July 2021. So we have selected Kerala as patch 1, two neighbouring states of Kerala- Maharashtra as patch 2, and Tamil Nadu as patch 3. We have used the data sets of active COVID-19 cases^47^ from July 14th to August 20th, 2021. Since the active individuals are those who are clinically reported as COVID-19 patients, the *H* compartment of our model is fitted with the data (Fig. 2a,c,e). We estimate the parameters $\beta _{s_i}$, the effective contact rate between susceptible and symptomatic, $\gamma _i$, quarantined rate of asymptomatic individuals, and $\alpha _{s_i}$, quarantined rate of symptomatic individuals, which are given in Table 4 for all the three patches. By looking at the estimated values, it is observed that the effective contact rate between susceptible and symptomatic is quite high, and the quarantined rate of symptomatic is quite low in patch 1. Whereas in patch 3 contact rate between susceptible and symptomatic is very low, and the quarantined rate of symptomatic is high. The quarantined rate of asymptomatic is very low in patch 2. Therefore, patch 1 is considered the high prevalence state, and patch 3 is considered the lowest prevalence state among these three patches. With the help of the estimated parametric values, we have predicted the number of active cases in all three patches for the next 22 days, i.e., from August 21st to September 11th, 2021. It is observed that the active cases graph is decaying in all patches in Fig. 2b,d,f.

We further explore the impact of mobility of COVID-19 spread, choosing different mobility rates between patches. It is observed that when there is mobility restriction between Kerala and either Maharashtra or Tamil Nadu, the number of active cases increases in Kerala (Fig. 4) and decreases in other states. Therefore, it is noted that when mobility is prohibited from the high prevalence state to lower prevalence states, the number of active cases number will increase in the high prevalence state. A similar result is seen if the immigration rate is higher than the emigration rate in the high-prevalence state. However, an increase in mobility rates reduce the disease prevalence in high prevalence state (Kerala) and increase disease outbreak in lower prevalence states (Maharashtra, Tamil Nadu) (Fig. 3). Also, the number of active cases will decrease in the high prevalence state and increase in the lower prevalence states if the emigration rate is higher than the immigration rate in the high prevalence state.

We also exhibit the impact of mobility on COVID-19 disease extinction (Fig. 4). For this, we have reduced the effective contact rate between susceptible and symptomatic in the lower prevalence states, Maharashtra and Tamil Nadu. It is later shown that the disease becomes extinct in the lower prevalence states (Maharashtra and Tamil Nadu) if all patches are isolated from each other, that is, no immigration or emigration will happen in any patch. In this case, the disease will persist in only the high prevalence state Kerala (Fig. 4).

## Conclusion

When COVID-19 first emerged in 2020, we observed that every nation had its own set of travel restrictions in place due to the human-to-human transmission of this disease. The movement of individuals from one location to another played a huge role in the spread of disease worldwide. Similar to other countries, the Indian government had imposed several travel restrictions at all levels including both domestic and international travel. India is a huge country with a large population in each of its states. Every day, a significantly large number of people move from one state to another, which is why this movement of people was crucial in the spread of the COVID-19 disease in India. As mentioned earlier, we included the mobility factor and our research demonstrated that if mobility begins in every patch (state in India), the three-patch system transforms into an epidemic system, and disease will persist in every patch.

Therefore, the COVID-19 disease outbreak is influenced by the movement of the population in a big way. Herein, it is noted that based on the mobility restrictions the basic reproduction number can increase or decrease, although all rate parameters are fixed. This research offers significant results from numerical experiments that are employed as a specific control.

Although the presented results have been confined to only three Indian states, the outcomes are scalable to an even larger number of states. Additionally, similar findings can be obtained for multiple countries using this methodology. E.g. European Union has a comparable demographic zone, making it possible to utilize the present model as a model for other European nations. We have considered the three Indian states as an example and shown the impact of various parameters related to mobility on the spread and control of the disease. Nonetheless, our main results are generic in nature and are independent of the location. The proper setting of parameters helps in predicting the course of disease in any country or state/s.

### Importance of mobility restrictions

 Various variants of COVID-19 are detected, thus by imposing mobility restrictions, we can mitigate the introduction and spread of new variants. Mobility restrictions can reduce the transmission of infection by limiting the mobility of infective people across borders. This offers the public health administration a crucial time window to adopt and improve upon domestic control measures like testing, contact tracing, quarantine regulations, and vaccination campaigns, etc. Each country/patch has a limited capacity for treatment. So, mobility restrictions can assist in reducing the additional burden on their healthcare infrastructure. Moreover, it is easy to contain and control disease in a smaller patch rather than in the whole population. Hence, if there is a surge in a particular location (patch), it is advisable to isolate that patch.

### Future direction

Presented study needs to be scaled up to a larger population where one can investigate COVID-19 distribution in different countries rather than states and analyse the impact of international travel restrictions on disease control. We plan to incorporate the impact of vaccination too in our future work.

## Supplementary Information

Supplementary Information.

## Data Availability

The datasets analysed during the current study are available in the COVID-19 India repository “https://www.covid19india.org/”.
