# Supplementary material for: Role of immigration and emigration on the spread of COVID-19 in a multipatch environment: a case study of India
Source: Sci Rep. 2023 Jun 29;13:10546. doi: 10.1038/s41598-023-37192-z (PMC10310821; doi:10.1038/s41598-023-37192-z)
Supplement: Supplementary file 1 — Supplementary Information. [file 41598_2023_37192_MOESM1_ESM.pdf]

Supplementary document

Mathematically, we express our system with the help of the following system of differential equations.

$$
\begin{aligned}
\frac{dS_i}{dt} &= \Lambda_i - \mu_i S_i - \frac{\beta_{s_i}}{1+m_{s_i}I_{s_i}} S_i I_{s_i} - \frac{\beta_{a_i}}{1+m_{a_i}I_{a_i}} S_i I_{a_i} + \sum_{\substack{j=1,\\ j\neq i}}^{n} (a_{ij}S_j - a_{ji}S_i),\\
\frac{dE_i}{dt} &= \frac{\beta_{s_i}}{1+m_{s_i}I_{s_i}} S_i I_{s_i} + \frac{\beta_{a_i}}{1+m_{a_i}I_{a_i}} S_i I_{a_i} - (\sigma_i + \mu_i)E_i + \sum_{\substack{j=1,\\ j\neq i}}^{n} (b_{ij}E_j - b_{ji}E_i),\\
\frac{dI_{a_i}}{dt} &= \varepsilon_i \sigma_i E_i - \mu_i I_{a_i} - \gamma_i I_{a_i} - \theta_i I_{a_i} + \sum_{\substack{j=1,\\ j\neq i}}^{n} (c_{ij}I_{a_j} - c_{ji}I_{a_i}),\\
\frac{dQ_i}{dt} &= \gamma_i I_{a_i} - (\eta_i + \rho_i + \mu_i)Q_i,\\
\frac{dI_{s_i}}{dt} &= (1-\varepsilon_i)\sigma_i E_i - (\mu_{s_i} + \mu_i)I_{s_i} + \eta_i Q_i + \theta_i I_{a_i} - \alpha_{s_i} I_{s_i} - \frac{\alpha_i I_{s_i}}{1+\nu_i I_{s_i}},\\
\frac{dH_i}{dt} &= \alpha_{s_i} I_{s_i} + \frac{\alpha_i I_{s_i}}{1+\nu_i I_{s_i}} - (\mu_{h_i} + \mu_i)H_i - \xi_i H_i,\\
\frac{dR_i}{dt} &= \rho_i Q_i + \xi_i H_i - \mu_i R_i + \sum_{\substack{j=1,\\ j\neq i}}^{n} (d_{ij}R_j - d_{ji}R_i).
\end{aligned}
\tag{1}
$$

1 Proof of results in subsection 3.1 (i.e., case 1: both immigration and emigration are allowed)

1.1 Analysis of the disease-free equilibrium (DFE)

Consider that system (1) has disease-free equilibrium in Γ then for each i $(i = 1, 2, \ldots, n)$ $E_0^{(i)}(S_{i_0}, 0, 0, 0, 0, 0, 0)$ satisfies following equations:

$$
\begin{cases}
\Lambda_i - \mu_i S_{i_0} + \sum_{\substack{j=1,\\ j\neq i}}^{n} (a_{ij}S_{j_0} - a_{ji}S_{i_0}) = 0,\\
-(\sigma_i + \mu_i)E_i + \sum_{\substack{j=1,\\ j\neq i}}^{n} (b_{ij}E_j - b_{ji}E_i) = 0,\\
\varepsilon_i \sigma_i E_i = 0,\\
-(\eta_i + \rho_i + \mu_i)Q_i = 0,\\
(1-\varepsilon_i)\sigma_i E_i + \eta_i Q_i = 0,\\
-(\mu_{h_i} + \mu_i)H_i - \xi_i H_i = 0,\\
\rho_i Q_i + \xi_i H_i - \mu_i R_i + \sum_{\substack{j=1,\\ j\neq i}}^{n} (d_{ij}R_j - d_{ji}R_i) = 0.
\end{cases}
\tag{2}
$$

Which gives $E_i = 0$, $Q_i = 0$, $H_i = 0$ and

$$
\Lambda_i - \mu_i S_{i_0} + \sum_{\substack{j=1,\\ j\neq i}}^{n} (a_{ij}S_{j_0} - a_{ji}S_{i_0}) = 0, \tag{3}
$$

$$
-\mu_i R_i + \sum_{\substack{j=1,\\ j\neq i}}^{n} (d_{ij}R_j - d_{ji}R_i) = 0, \tag{4}
$$

for $i = 1,2,\ldots,n$. Equations (3) and (4) can be written as $G_0 S_0 = b$ and $G_1 R = 0$, respectively, where $S_0 = [S_{i_0}]_{n\times 1}$, $b = [\Lambda_i]_{n\times 1}$, $R = [R_i]_{n\times 1}$, and

$$G_0 = [\delta_{ij}(\mu_i + \sum_{\substack{j=1,\\ j\neq i}}^{n} a_{ji}) - (1-\delta_{ij})a_{ij}]_{n\times n}, \tag{5}$$

$$G_1 = [\delta_{ij}(\mu_i + \sum_{\substack{j=1,\\ j\neq i}}^{n} d_{ji}) - (1-\delta_{ij})d_{ij}]_{n\times n}. \tag{6}$$

Here G_1 is also nonsingular $\mathscr{M}$- matrix and therefore G_1^{-1} is non negative. Thus we get $S_0 = (G_0^{-1}b)_{n\times 1}$ and $R = 0$. Hence, system (1) has a disease-free equilibrium point, $E_0^{(i)} = (S_{i_0},0,0,0,0,0,0) = ((G_0^{-1}b)_i,0,0,0,0,0,0) \in \mathbb{R}_+^7$ for each i ($i = 1,2,\ldots,n$). As each $E_0^{(i)}$ is independent, the disease-free equilibrium for system (1) can be obtained as $E_0^{(multi)}$.

1.2 The basic reproduction number

The infection carrying state variables for the system (1) are $E_1,E_2,\ldots,E_n,I_{a_1},I_{a_2},\ldots,I_{a_n},Q_1,Q_2,\ldots,Q_n,I_{s_1},I_{s_2},\ldots,I_{s_n}$.

Following lemma 1[1] we construct, $\mathscr{F} = \begin{bmatrix}\mathscr{F}_1\\ \mathbf{0}\\ \mathbf{0}\\ \mathbf{0}\end{bmatrix}_{4n\times 1}$ and $\mathscr{V} = \begin{bmatrix}\mathscr{V}_1\\ \mathscr{V}_2\\ \mathscr{V}_3\\ \mathscr{V}_4\end{bmatrix}_{4n\times 1}$,

where $\mathbf{0} = [0]_{n\times 1}$,

$$\begin{aligned}
\mathscr{F}_1 &= [\frac{\beta_{s_i}}{1+m_{s_i}I_{s_i}}S_i I_{s_i} + \frac{\beta_{a_i}}{1+m_{a_i}I_{a_i}}S_i I_{a_i}]_{n\times 1},\\
\mathscr{V}_1 &= [(\sigma_i+\mu_i)E_i - \sum_{\substack{j=1,\\ j\neq i}}^{n}(b_{ij}E_j - b_{ji}E_i)]_{n\times 1},\\
\mathscr{V}_2 &= [-\varepsilon_i\sigma_i E_i + (\mu_i+\gamma_i+\theta_i)I_{a_i} - \sum_{\substack{j=1,\\ j\neq i}}^{n}(c_{ij}I_{a_j} - c_{ji}I_{a_i})]_{n\times 1},\\
\mathscr{V}_3 &= [-\gamma_i I_{a_i} + (\eta_i+\rho_i+\mu_i)Q_i]_{n\times 1},\\
\mathscr{V}_4 &= [-(1-\varepsilon_i)\sigma_i E_i + (\mu_{s_i}+\mu_i)I_{s_i} - \eta_i Q_i + \alpha_{s_i}I_{s_i} + \frac{\alpha_i I_{s_i}}{1+\nu_i I_{s_i}} - \theta_i I_{a_i}]_{n\times 1}.
\end{aligned}$$

At the disease-free equilibrium $E_0^{(multi)}$, we get,

$$F = \begin{bmatrix}\mathbf{0} & F_{12} & \mathbf{0} & F_{14}\\ \mathbf{0} & \mathbf{0} & \mathbf{0} & \mathbf{0}\\ \mathbf{0} & \mathbf{0} & \mathbf{0} & \mathbf{0}\\ \mathbf{0} & \mathbf{0} & \mathbf{0} & \mathbf{0}\end{bmatrix}_{4n\times 4n}, \quad \text{and} \quad V = \begin{bmatrix}V_{11} & \mathbf{0} & \mathbf{0} & \mathbf{0}\\ V_{21} & V_{22} & \mathbf{0} & \mathbf{0}\\ \mathbf{0} & V_{32} & V_{33} & \mathbf{0}\\ V_{41} & V_{42} & V_{43} & V_{44}\end{bmatrix}_{4n\times 4n}. \tag{7}$$

where, $\mathbf{0} = [0]_{n\times n}$ and

$$\begin{cases}
F_{12} = [\delta_{ij}\beta_{a_i}S_{i_0}]_{n\times n}, \quad F_{14} = [\delta_{ij}\beta_{s_i}S_{i_0}]_{n\times n},\\
V_{11} = [\delta_{ij}(\sigma_i+\mu_i+\sum_{\substack{j=1,\\ j\neq i}}^{n} b_{ji}) - (1-\delta_{ij})b_{ij}]_{n\times n},\\
V_{21} = [-\delta_{ij}\varepsilon_i\sigma_i]_{n\times n},\\
V_{22} = [\delta_{ij}(\mu_i+\gamma_i+\theta_i+\sum_{\substack{j=1,\\ j\neq i}}^{n} c_{ji}) - (1-\delta_{ij})c_{ij}]_{n\times n},\\
V_{32} = [-\delta_{ij}\gamma_i]_{n\times n}, \quad V_{33} = [\delta_{ij}(\eta_i+\rho_i+\mu_i)]_{n\times n},\\
V_{41} = [-\delta_{ij}(1-\varepsilon_i)\sigma_i]_{n\times n}, \quad V_{42} = [-\delta_{ij}\theta_i]_{n\times n},\\
V_{43} = [-\delta_{ij}\eta_i]_{n\times n}, \quad V_{44} = [\delta_{ij}(\mu_{s_i}+\mu_i+\alpha_i+\alpha_{s_i})]_{n\times n}.
\end{cases} \tag{8}$$

It is easily seen that F is non negative and V is nonsingular matrix and spectral radius of matrix FV^{-1} is the required basic reproduction number $(\mathscr{R}_0^{(multi)})$[1]. Thus, $\mathscr{R}_0^{(multi)} = \rho(FV^{-1}) = \rho(-(F_{12}V_{21} + F_{14}(V_{21}(V_{32}V_{43} - V_{33}V_{42}) + V_{41}V_{22}V_{33})V_{33}^{-1}V_{44}^{-1})V_{11}^{-1}V_{22}^{-1})$.

1.3 Global stability of DFE

To show the global stability property of $E_0^{(multi)}$ we construct the following Lyapunov function,

$$L_1 = \sum_{i=1}^{n} (k_{1_i} E_i + k_{2_i} I_{a_i} + k_{3_i} Q_i + k_{4_i} I_{s_i}),$$

where $k_{m_i} > 0$, $i = 1,2,\ldots,n$, $m = 1,2,3,4$. Following the calculations similarly as given by Meng and Zhu[2], it is obtained that matrix $V^{-1}F$ has a positive left eigenvector $z = (l_{1_1}, l_{1_2}, \ldots, l_{1_n}, l_{2_1}, l_{2_2}, \ldots, l_{2_n}, l_{3_1}, l_{3_2}, \ldots, l_{3_n}, l_{4_1}, l_{4_2}, \ldots, l_{4_n})$ with respect to the eigen value $\rho(V^{-1}F)$. Thus,

$$zV^{-1}F = \rho(V^{-1}F)z.$$

Assume that $(k_{1_1}, k_{1_2}, \ldots, k_{1_n}, k_{2_1}, k_{2_2}, \ldots, k_{2_n}, k_{3_1}, k_{3_2}, \ldots, k_{3_n}, k_{4_1}, k_{4_2}, \ldots, k_{4_n}) = zV^{-1}$. Then, the derivative of L_1 along the solutions trajectory of system (1) becomes,

$$\begin{aligned}
\frac{dL_1}{dt} =& \sum_{i=1}^{n} (k_{1_i} \frac{dE_i}{dt} + k_{2_i} \frac{dI_{a_i}}{dt} + k_{3_i} \frac{dQ_i}{dt} + k_{4_i} \frac{dI_{s_i}}{dt}), \\
=& \sum_{i=1}^{n} k_{1_i} \left\{ \frac{\beta_{s_i}}{1+m_{s_i} I_{s_i}} S_i I_{s_i} + \frac{\beta_{a_i}}{1+m_{a_i} I_{a_i}} S_i I_{a_i} - (\sigma_i + \mu_i) E_i + \sum_{\substack{j=1,\\ j\neq i}}^{n} (b_{ij} E_j - b_{ji} E_i) \right\} + \\
& \sum_{i=1}^{n} k_{2_i} \left\{ \varepsilon_i \sigma_i E_i - (\mu_i + \gamma_i + \theta_i) I_{a_i} + \sum_{\substack{j=1,\\ j\neq i}}^{n} (c_{ij} I_{a_j} - c_{ji} I_{a_i}) \right\} + \sum_{i=1}^{n} k_{3_i} \{ \gamma_i I_{a_i} - (\eta_i + \rho_i + \mu_i) Q_i \} \\
& + \sum_{i=1}^{n} k_{4_i} \left\{ (1-\varepsilon_i)\sigma_i E_i - (\mu_{s_i} + \mu_i + \frac{\alpha_i}{1+\nu_i I_{s_i}}) I_{s_i} + \eta_i Q_i + \theta_i I_{a_i} \right\}, \\
\leq & \sum_{i=1}^{n} k_{1_i} \left\{ \beta_{s_i} S_{i_0} I_{s_i} + \beta_{a_i} S_{i_0} I_{a_i} - (\sigma_i + \mu_i) E_i + \sum_{\substack{j=1,\\ j\neq i}}^{n} (b_{ij} E_j - b_{ji} E_i) \right\} + \sum_{i=1}^{n} k_{2_i} \\
& \left\{ \varepsilon_i \sigma_i E_i - (\mu_i + \gamma_i + \theta_i) I_{a_i} + \sum_{\substack{j=1,\\ j\neq i}}^{n} (c_{ij} I_{a_j} - c_{ji} I_{a_i}) \right\} + \sum_{i=1}^{n} k_{3_i} \{ \gamma_i I_{a_i} - (\eta_i + \rho_i + \mu_i) Q_i \} + \\
& \sum_{i=1}^{n} k_{4_i} \left\{ (1-\varepsilon_i)\sigma_i E_i - (\mu_{s_i} + \mu_i + \alpha_i + \alpha_{s_i}) I_{s_i} + \frac{\alpha_i \nu_i I_{s_i}}{1+\nu_i I_{s_i}} + \eta_i Q_i + \theta_i I_{a_i} \right\}, \\
=& (k_{1_1}, k_{1_2}, \ldots, k_{1_n})(-V_{11} E + F_{12} I_a + F_{14} I_s) - (k_{2_1}, k_{2_2}, \ldots, k_{2_n})(V_{21} E + V_{22} I_a) - (k_{3_1}, k_{3_2}, \ldots, k_{3_n})(V_{32} I_a + V_{33} Q) \\
& - (k_{4_1}, k_{4_2}, \ldots, k_{4_n})(V_{41} E + V_{42} I_a + V_{43} Q + V_{44} I_s) + \sum_{i=1}^{n} \frac{k_{4_i} \alpha_i \nu_i I_{s_i}}{1+\nu_i I_{s_i}}, \quad (\forall F_{ij}, V_{ij},\ i,j = 1,2,3,4, \text{ as defined in (8)}), \\
=& (k_{1_1}, k_{1_2}, \ldots, k_{1_n}, k_{2_1}, k_{2_2}, \ldots, k_{2_n}, k_{3_1}, k_{3_2}, \ldots, k_{3_n}, k_{4_1}, k_{4_2}, \ldots, k_{4_n})(F - V)X + \sum_{i=1}^{n} \frac{k_{4_i} \alpha_i \nu_i I_{s_i}}{1+\nu_i I_{s_i}}, \\
=& (l_{1_1}, l_{1_2}, \ldots, l_{1_n}, l_{2_1}, l_{2_2}, \ldots, l_{2_n}, l_{3_1}, l_{3_2}, \ldots, l_{3_n}, l_{4_1}, l_{4_2}, \ldots, l_{4_n}) V^{-1}(F - V)X + \sum_{i=1}^{n} \frac{k_{4_i} \alpha_i \nu_i I_{s_i}}{1+\nu_i I_{s_i}}, \\
=& (l_{1_1}, l_{1_2}, \ldots, l_{1_n}, l_{2_1}, l_{2_2}, \ldots, l_{2_n}, l_{3_1}, l_{3_2}, \ldots, l_{3_n}, l_{4_1}, l_{4_2}, \ldots, l_{4_n})(V^{-1}F - I)X + \sum_{i=1}^{n} \frac{k_{4_i} \alpha_i \nu_i I_{s_i}}{1+\nu_i I_{s_i}}, \\
=& (l_{1_1}, l_{1_2}, \ldots, l_{1_n}, l_{2_1}, l_{2_2}, \ldots, l_{2_n}, l_{3_1}, l_{3_2}, \ldots, l_{3_n}, l_{4_1}, l_{4_2}, \ldots, l_{4_n})(\mathscr{R}_0^{(multi)} - 1)X + \sum_{i=1}^{n} \frac{k_{4_i} \alpha_i \nu_i I_{s_i}}{1+\nu_i I_{s_i}},
\end{aligned}$$

taking $X = (E_1, E_2, \ldots, E_n, I_{a_1}, I_{a_2}, \ldots, I_{a_n}, Q_1, Q_2, \ldots, Q_n, I_{s_1}, I_{s_2}, \ldots, I_{s_n})^T$. So, $\frac{dL_1}{dt} < 0$ if $\mathscr{R}_0^{(multi)} < 1$ and $\nu_i = 0$. Further, $\frac{dL_1}{dt} = 0$ if $E_i = I_{a_i} = Q_i = I_{s_i} = 0$ for $i = 1,2,\ldots,n$. Again $E_i = I_{a_i} = Q_i = I_{s_i} = 0$ imply $R_i = 0$ and $S_i = S_{i_0}$ for $i = 1,2,\ldots,n$. Thus, the singleton set $\{E_0^{(multi)}\}$ is the largest positive invariant set contained in $\{(S_1, E_1, I_{a_1}, Q_1, I_{s_1}, H_1, R_1,$

$\dots, S_n, E_n, I_{a_n}, Q_n, I_{s_n}, H_n, R_n) \in \Gamma : \frac{dL_1}{dt} = 0\}$. Therefore, by Lyapunov LaSalle's theorem[3], $E_0^{(multi)}$ is globally asymptotically stable in the interior of Γ for $\mathscr{R}_0^{(multi)} < 1$ and $\nu_i = 0$ for $i = 1, 2, \dots, n$.

1.4 Existence of endemic equilibrium

As we proved that the unique DFE $E_0^{(multi)}$ is locally stable for $\mathscr{R}_0^{(multi)} < 1$ and is unstable for $\mathscr{R}_0^{(multi)} > 1$ in the basic feasible region Γ. According to the Theorem 2.2[4] the instability of E_0 for $\mathscr{R}_0^{(multi)} > 1$ confirms the uniform persistence of the system (1), which implies the persistence of endemic equilibrium point in the positive invariant set Γ for $\mathscr{R}_0^{(multi)} > 1$ (Theorem D.3[5]). Hence, we can say that the endemic equilibrium point $E_*^{(multi)}$ always exists for $\mathscr{R}_0^{(multi)} > 1$. Similar proof may be followed as provided by Rebaza[6].

Now we establish possibility of existence of endemic equilibrium for $\mathscr{R}_0^{(multi)} < 1$. For this, assume that the multi-patch system (1) has an endemic equilibrium point in Γ: $E_*^{(multi)} = (S_{1_*}, E_{1_*}, I_{a_{1_*}}, Q_{1_*}, I_{s_{1_*}}, H_{1_*}, R_{1_*}, \dots, S_{n_*}, E_{n_*}, I_{a_{n_*}}, Q_{n_*}, I_{s_{n_*}}, H_{n_*}, R_{n_*}) \in \mathbb{R}_+^{7n}$, where $S_{i_*}, E_{i_*}, I_{a_{i_*}}, Q_{i_*}, I_{s_{i_*}}, H_{i_*}, R_{i_*}$ satisfy following equations for $i = 1, 2, \dots, n$,

$$\Lambda_i - \mu_i S_{i_*} - \frac{\beta_{s_i}}{1 + m_{s_i} I_{s_{i_*}}} S_{i_*} I_{s_{i_*}} - \frac{\beta_{a_i}}{1 + m_{a_i} I_{a_{i_*}}} S_{i_*} I_{a_{i_*}} + \sum_{\substack{j=1,\\ j\neq i}}^{n} (a_{ij} S_{j_*} - a_{ji} S_{i_*}) = 0, \tag{9a}$$

$$\frac{\beta_{s_i}}{1 + m_{s_i} I_{s_{i_*}}} S_{i_*} I_{s_{i_*}} + \frac{\beta_{a_i}}{1 + m_{a_i} I_{a_{i_*}}} S_{i_*} I_{a_{i_*}} - (\sigma_i + \mu_i) E_{i_*} + \sum_{\substack{j=1,\\ j\neq i}}^{n} (b_{ij} E_{j_*} - b_{ji} E_{i_*}) = 0, \tag{9b}$$

$$\varepsilon_i \sigma_i E_{i_*} - \mu_i I_{a_{i_*}} - \gamma_i I_{a_{i_*}} - \theta_i I_{a_{i_*}} + \sum_{\substack{j=1,\\ j\neq i}}^{n} (c_{ij} I_{a_{j_*}} - c_{ji} I_{a_{i_*}}) = 0, \tag{9c}$$

$$\gamma_i I_{a_{i_*}} - (\eta_i + \rho_i + \mu_i) Q_{i_*} = 0, \tag{9d}$$

$$(1 - \varepsilon_i) \sigma_i E_{i_*} - (\mu_{s_i} + \mu_i) I_{s_{i_*}} + \eta_i Q_{i_*} + \theta_i I_{a_{i_*}} - \alpha_{s_i} I_{s_{i_*}} - \frac{\alpha_i I_{s_{i_*}}}{1 + \nu_i I_{s_{i_*}}} = 0, \tag{9e}$$

$$\alpha_{s_i} I_{s_{i_*}} + \frac{\alpha_i I_{s_{i_*}}}{1 + \nu_i I_{s_{i_*}}} - (\mu_{h_i} + \mu_i) H_{i_*} - \xi_i H_{i_*} = 0, \tag{9f}$$

$$\rho_i Q_{i_*} + \xi_i H_{i_*} - \mu_i R_{i_*} + \sum_{\substack{j=1,\\ j\neq i}}^{n} (d_{ij} R_{j_*} - d_{ji} R_{i_*}) = 0. \tag{9g}$$

Denote, $S_* = [S_{i_*}]_{n\times 1}$, $E_* = [E_{i_*}]_{n\times 1}$, $I_{a_*} = [I_{a_{i_*}}]_{n\times 1}$, $Q_* = [Q_{i_*}]_{n\times 1}$, $I_{s_*} = [I_{s_{i_*}}]_{n\times 1}$, $H_* = [H_{i_*}]_{n\times 1}$, and $R_* = [R_{i_*}]_{n\times 1}$. From the equation (9c)-(9d) we have,

$$E_{i_*} = \frac{(\mu_i + \gamma_i + \theta_i + \sum_{\substack{j=1,\\ j\neq i}}^{n} c_{ji}) I_{a_{i_*}} - \sum_{\substack{j=1,\\ j\neq i}}^{n} c_{ij} I_{a_{j_*}}}{\varepsilon_i \sigma_i}, \quad Q_{i_*} = \frac{\gamma_i}{\eta_i + \rho_i + \mu_i} I_{a_{i_*}}, \; i = 1, 2, \dots, n.$$

So, $E_* = -V_{22} V_{21}^{-1} I_{a_*}$ and $Q_* = -V_{32} V_{33}^{-1} I_{a_*}$. Now, equation (9e) can be rewritten as,

$$(V_{44} - G_2^*) I_{s_*} = V_3 I_{a_*}, \tag{10}$$

where $V_3 = (V_{21}(V_{32}V_{43} - V_{33}V_{42}) + V_{41}V_{22}V_{33}) V_{33}^{-1} V_{21}^{-1}$, and $G_2^* = [\delta_{ij}(\alpha_i \nu_i \frac{I_{s_{i_*}}}{1 + \nu_i I_{s_{i_*}}})]_{n\times n}$.

Using equation (9b) we have for $i = 1, 2, \dots, n$,

$$\frac{\beta_{s_i}}{1 + m_{s_i} I_{s_{i_*}}} S_{i_*} I_{s_{i_*}} + \frac{\beta_{a_i}}{1 + m_{a_i} I_{a_{i_*}}} S_{i_*} I_{a_{i_*}} = (\sigma_i + \mu_i + \sum_{\substack{j=1,\\ j\neq i}}^{n} b_{ji}) E_{i_*} - \sum_{\substack{j=1,\\ j\neq i}}^{n} b_{ij} E_{j_*}.$$

Therefore, $G_3^* S_* = V_{11} E_*$, where $G_3^* = [\delta_{ij}(\frac{\beta_{s_i} I_{s_{i_*}}}{1 + m_{s_i} I_{s_{i_*}}} + \frac{\beta_{a_i} I_{a_{i_*}}}{1 + m_{a_i} I_{a_{i_*}}})]_{n\times n}$. With the help of (5) and $b = [\Lambda_i]_{n\times 1}$, equation (9a) can

be expressed as follows

$$
\begin{aligned}
&b - G_0 S_* - V_{11} E_* = 0,\\
&G_0^{-1} b - S_* - G_0^{-1} V_{11} E_* = 0,\\
&S_0 - S_* - G_0^{-1} V_{11} E_* = 0,\\
&G_4^* G_3^* S_0 - G_4^* G_3^* G_3^{*-1} V_{11} E_* - G_4^* G_3^* G_0^{-1} V_{11} E_* = 0,\\
&V_3 V_{21} V_{11}^{-1} V_{22}^{-1} V_{44}^{-1} G_4^* G_3^* S_0 + V_3 V_{44}^{-1} G_4^* I_a^* + V_3 V_{44}^{-1} G_0^{-1} G_4^* G_3^* I_{a_*} = 0.
\end{aligned} \tag{11}
$$

We denote $D[I_{s_{i*}}] = [\delta_{ij} I_{s_{i*}}]_{n\times n}$. After evaluation, we obtain

$$V_3 G_4^* G_3 S_0 = V_3 F_{14} + F_{12} V_{44} + M_{11}.D[I_{s_{i*}}] + M_{12}.D[I_{s_{i*}}^2] + M_{13}.D[I_{s_{i*}}^3], \tag{12}$$

$$V_3 G_4^* I_{a_*} = V_{44} + M_{21}.D[I_{s_{i*}}] + M_{22}.D[I_{s_{i*}}^2] + M_{23}.D[I_{s_{i*}}^3] + M_{24}.D[I_{s_{i*}}^4], \tag{13}$$

$$V_3 G_4^* G_3 I_{a_*} = M_{31}.D[I_{s_{i*}}] + M_{32}.D[I_{s_{i*}}^2] + M_{33}.D[I_{s_{i*}}^3] + M_{34}.D[I_{s_{i*}}^4]. \tag{14}$$

where,

$$
\begin{aligned}
M_{11} =& 2V_3 F_{14} D[\nu_i] + V_{44}(F_{14} D[m_{a_i}] + F_{12} D[m_{s_i} + 2\nu_i]) - F_{12} D[\alpha_i \nu_i],\\
M_{12} =& V_3 F_{14} D[\nu_i^2] + V_{44}(F_{14} 2D[m_{a_i}\nu_i] + F_{12} D[\nu_i(2m_{s_i} + \nu_i)] - (F_{14} D[m_{a_i}\alpha_i \nu_i] + F_{12} D[\alpha_i \nu_i (m_{s_i} + \nu_i)],\\
M_{13} =& V_{44}(F_{14} D[m_{a_i}\nu_i^2] + F_{12} D[m_{s_i}\nu_i^2]) - (F_{14} D[m_{a_i}\alpha_i \nu_i^2] + F_{12} D[\alpha_i \nu_i^2 m_{s_i}]),\\
M_{21} =& V_{44} D[m_{s_i} + 2\nu_i] - D[\alpha_i \nu_i],\\
M_{22} =& V_{44} D[\nu_i(2m_{s_i} + \nu_i)] + V_3^{-1} V_{44}\{V_{44} D[m_{a_i}(m_{s_i} + 2\nu_i)] - 2D[m_{a_i}\alpha_i \nu_i]\} - D[\alpha_i \nu_i (m_{s_i} + \nu_i)],\\
M_{23} =& V_{44} D[m_{s_i}\nu_i^2] + V_3^{-1} V_{44}\{V_{44} D[m_{a_i}\nu_i(2m_{s_i} + \nu_i)] - 2D[m_{a_i}\alpha_i \nu_i (m_{s_i} + \nu_i)]\} - V_3^{-1} D[\alpha_i^2 \nu_i^2 (m_{a_i} - m_{s_i})],\\
M_{24} =& V_3^{-1} D[m_{s_i} m_{a_i} \nu_i^2](V_{44}^2 - 2V_{44} D[\alpha_i] + D[\alpha_i^2]),\\
M_{31} =& V_{44} D[\beta_{s_i}] - D[\beta_{s_i}] + V_3^{-1} V_{44}^2 D[\beta_{a_i}],\\
M_{32} =& 2V_{44} D[\beta_{s_i}\nu_i] - D[\beta_{s_i}\nu_i] + V_3^{-1} V_{44}^2 (D[\beta_{s_i} m_{a_i}] + D[\beta_{a_i}(m_{s_i} + 2\nu_i)]) - 2V_3^{-1} V_{44} D[\beta_{a_i}\alpha_i \nu_i],\\
M_{33} =& V_{44} D[\beta_{s_i}\nu_i^2] + V_3^{-1} V_{44}^2 (2D[\beta_{s_i} m_{2_i} \nu_i] + D[\beta_{a_i}\nu_i(2m_{s_i} + \nu_i)]) - 2V_3^{-1} V_{44}(D[\beta_{s_i} m_{a_i}\alpha_i \nu_i] + D[\beta_{a_i}\alpha_i \nu_i (m_{s_i} + \nu_i)])\\
&+ V_3^{-1} D[\beta_{a_i}\alpha_i^2 \nu_i^2],\\
M_{34} =& V_3^{-1} D[(\beta_{s_i} m_{a_i} + \beta_{a_i} m_{s_i})\nu_i^2](V_{44}^2 - 2V_{44} D[\alpha_i] + D[\alpha_i^2]).
\end{aligned}
$$

Putting (12)- (14) in (11) we have,

$$
\begin{aligned}
&(F_{14} V_3 V_{21} V_{11}^{-1} V_{22}^{-1} V_{44}^{-1} + F_{12} V_{21} V_{11}^{-1} V_{22}^{-1} + I_{n\times n} + V_{44}^{-1}(V_{21} V_{11}^{-1} V_{22}^{-1} M_{11} + M_{21} + G_0^{-1} M_{31}) D[I_{s_{i*}}] + V_{44}^{-1}(V_{21} V_{11}^{-1} V_{22}^{-1} M_{12}\\
&+ M_{22} + G_0^{-1} M_{32}) D[I_{s_{i*}}^2] + V_{44}^{-1}(V_{21} V_{11}^{-1} V_{22}^{-1} M_{13} + M_{23} + G_0^{-1} M_{33}) D[I_{s_{i*}}^3] + V_{44}^{-1}(M_{24} + G_0^{-1} M_{34}) D[I_{s_{i*}}^4]) I_{s_*} = 0,
\end{aligned}
$$

which implies,

$$(A_0 + A_1 D[I_{s_{i*}}] + A_2 D[I_{s_{i*}}^2] + A_3 D[I_{s_{i*}}^3] + A_4 D[I_{s_{i*}}^4]) I_{s_*} = 0, \tag{15}$$

where, $A_0 = I_{n\times n} - V^{-1} F$, $A_1 = V_{44}^{-1}(V_{21} V_{11}^{-1} V_{22}^{-1} M_{11} + M_{21} + G_0^{-1} M_{31})$, $A_2 = V_{44}^{-1}(V_{21} V_{11}^{-1} V_{22}^{-1} M_{12} + M_{22} + G_0^{-1} M_{32})$, $A_3 = V_{44}^{-1}(V_{21} V_{11}^{-1} V_{22}^{-1} M_{13} + M_{23} + G_0^{-1} M_{33})$, $A_4 = V_{44}^{-1}(M_{24} + G_0^{-1} M_{34})$. Using the equation (9f) we have unique $H_{i_*} > 0$ and the equation,

$$(\mu_{h_i} + \mu_i + \xi_i) H_{i_*} = \alpha_{1_i} I_{s_{i*}} + \frac{\alpha_i I_{s_{i*}}}{1 + \nu_i I_{s_{i*}}},$$

has positive root H_i^* for each positive $I_{s_{i*}}$, for $i = 1, 2, \ldots, n$.

From equation (9g) we get

$$(\mu_i + \sum_{\substack{j=1,\\ j\neq i}}^{n} d_{ji}) R_{i_*} - \sum_{\substack{j=1,\\ j\neq i}}^{n} d_{ij} R_{j_*} = \rho_i Q_{i_*} + \xi_i H_{i_*}.$$

Then $R_* = (G_1^{-1} G_4^*)_{n\times 1}$, with $G_4^* = [\rho_i Q_{i_*} + \xi_i H_{i_*}]_{n\times 1}$, for $i = 1, 2, \ldots, n$. Since, solving the equation (15) and finding the real positive root(s) analytically is almost impossible, we can only conclude that the endemic equilibrium $E_*^{(multi)}$ exists if equation (15) has any positive real root.

2 Proof of results in subsection 3.2 (i.e., case 2: Only emigration is allowed)

2.1 Local stability of DFE

The characteristic equation at $\tilde{E}_0^{(i)}$ becomes

$$h(\lambda) := (\lambda + \mu_i + \sum_{\substack{j=1,\\ j\neq i}}^{n} a_{ji})(\lambda + \mu_{h_i} + \mu_i + \xi_i)(\lambda + \mu_i + \sum_{\substack{j=1,\\ j\neq i}}^{n} d_{ji})(\lambda^4 + B_3\lambda^3 + B_2\lambda^2 + B_1\lambda + B_0) = 0, \tag{16}$$

where $l_{i_1} = \mu_i + \mu_{s_i} + \alpha_{s_i} + \alpha_i$, $l_{i_6} = \sigma_i + \mu_i + \sum_{\substack{j=1,\\ j\neq i}}^{n} b_{ji}$, $l_{i_7} = \mu_i + \gamma_i + \theta_i + \sum_{\substack{j=1,\\ j\neq i}}^{n} c_{ji}$, $l_{i_8} = \eta_i + \rho_i + \mu_i$, and

$B_3 = l_{i_1} + l_{i_6} + l_{i_7} + l_{i_8}$,

$B_2 = l_{i_1}l_{i_6} + l_{i_1}l_{i_7} + l_{i_1}l_{i_8} + l_{i_6}l_{i_7} + l_{i_6}l_{i_8} + l_{i_7}l_{i_8} - \varepsilon_i\sigma_i\beta_{a_i}\tilde{S}_{i_0} - (1-\varepsilon_i)\sigma_i\beta_{s_i}\tilde{S}_{i_0} = l_{i_1}l_{i_6}(1 - \tilde{\mathscr{R}}_0^{(i)}) + l_{i_1}l_{i_7} + l_{i_1}l_{i_8} + l_{i_6}l_{i_7}(1 - \tilde{\mathscr{R}}_0^{(i)}) + l_{i_6}l_{i_8} + l_{i_7}l_{i_8} + \frac{\varepsilon_i\sigma_i\beta_{s_i}\tilde{S}_{i_0}\theta_i}{l_{i_1}} + \frac{\varepsilon_i\sigma_i\beta_{s_i}\tilde{S}_{i_0}\gamma_i\eta_i}{l_{i_1}l_{i_8}} + \frac{(1-\varepsilon_i)\sigma_i\beta_{s_i}\tilde{S}_{i_0}l_{i_7}}{l_{i_1}} + \frac{\varepsilon_i\sigma_i\beta_{a_i}\tilde{S}_{i_0}l_{i_1}}{l_{i_7}} + \frac{\varepsilon_i\sigma_i\beta_{s_i}\tilde{S}_{i_0}\gamma_i\eta_i}{l_{i_7}l_{i_8}} + \frac{\varepsilon_i\sigma_i\beta_{s_i}\tilde{S}_{i_0}\theta_i}{l_{i_7}}$,

$B_1 = l_{i_1}l_{i_6}l_{i_7} + l_{i_1}l_{i_6}l_{i_8} + l_{i_1}l_{i_7}l_{i_8} + l_{i_6}l_{i_7}l_{i_8} - \varepsilon_i\sigma_i\beta_{a_i}\tilde{S}_{i_0}(l_{i_1} + l_{i_8}) - \varepsilon_i\sigma_i\beta_{s_i}\tilde{S}_{i_0}\theta_i - (1-\varepsilon_i)\sigma_i\beta_{s_i}\tilde{S}_{i_0}(l_{i_7} + l_{i_8}) = l_{i_1}l_{i_6}l_{i_7}(1 - \tilde{\mathscr{R}}_0^{(i)}) + l_{i_1}l_{i_6}l_{i_8}(1 - \tilde{\mathscr{R}}_0^{(i)}) + l_{i_1}l_{i_7}l_{i_8} + l_{i_6}l_{i_7}l_{i_8}(1 - \tilde{\mathscr{R}}_0^{(i)}) + \frac{\varepsilon_i\sigma_i\beta_{s_i}\tilde{S}_{i_0}\gamma_i\eta_i}{l_{i_8}} + \frac{\varepsilon_i\sigma_i\beta_{s_i}\tilde{S}_{i_0}\theta_i l_{i_8}}{l_{i_1}} + \frac{\varepsilon_i\sigma_i\beta_{s_i}\tilde{S}_{i_0}\gamma_i\eta_i}{l_{i_1}} + \frac{(1-\varepsilon_i)\sigma_i\beta_{s_i}\tilde{S}_{i_0}l_{i_7}l_{i_8}}{l_{i_1}} + \frac{\varepsilon_i\sigma_i\beta_{a_i}\tilde{S}_{i_0}l_{i_1}l_{i_8}}{l_{i_7}} + \frac{\varepsilon_i\sigma_i\beta_{s_i}\tilde{S}_{i_0}\gamma_i\eta_i}{l_{i_7}} + \frac{\varepsilon_i\sigma_i\beta_{s_i}\tilde{S}_{i_0}\theta_i l_{i_8}}{l_{i_7}}$,

$B_0 = l_{i_1}l_{i_6}l_{i_7}l_{i_8}(1 - \tilde{\mathscr{R}}_0^{(i)})$.

Hence, the characteristic equation (16) contains roots having negative real part for $\tilde{\mathscr{R}}_0^{(i)} < 1$ and contains a root with positive real part for $\tilde{\mathscr{R}}_0^{(i)} > 1$.

2.2 Local stability of endemic equilibrium

In this case, characteristic roots at endemic equilibrium $\tilde{E}_*^{(i)}$ are obtained by

$$(\lambda + \mu_{h_i} + \mu_i + \xi_i)(\lambda + \mu_i + \sum_{\substack{j=1,\\ j\neq i}}^{n} d_{ji})(\lambda^5 + \tilde{b}_{i_4}\lambda^4 + \tilde{b}_{i_3}\lambda^3 + \tilde{b}_{i_2}\lambda^2 + \tilde{b}_{i_1}\lambda + \tilde{b}_{i_0}) = 0, \tag{17}$$

where coefficients $\tilde{b}_{i_4}$ - $\tilde{b}_{i_0}$ are given in the manuscript. Therefore, applying Routh-Hurwitz criteria, we note that all roots of the characteristic equation (17) have negative real parts if the mentioned condition (i.e., condition (7) in manuscript) in the manuscript holds.

2.3 Existence of backward bifurcation

It is noticed that the characteristic equation (16) contains one zero root for $\tilde{\mathscr{R}}_0^{(i)} = 1$. Hence the Jacobian matrix $J|_{\tilde{E}_0^{(i)}}$ at $\tilde{E}_0^{(i)}$ has one zero eigenvalue at $\tilde{\mathscr{R}}_0^{(i)} = 1$ (say for $\beta_{a_i} = \beta_{a_i}^*$). At $\beta_{a_i}^*$ matrix $J|_{\tilde{E}_0^{(i)}}$ has the right eigen vector $U_1 = (x_1, x_2, x_3, x_4, x_5, x_6, x_7)^T$ and left eigen vector $U_2 = (y_1, y_2, y_3, y_4, y_5, y_6, y_7)^T$ with respect to zero eigen value, where $x_1 = 1, x_2 = 1, x_3 = \frac{\varepsilon_i\sigma_i}{\mu_i + \gamma_i + \theta_i + \sum_{\substack{j=1,\\ j\neq i}}^{n} c_{ji}}$,

$x_4 = \frac{\gamma_i x_3}{\eta_i + \rho_i + \mu_i}$, $x_5 = \frac{\mu_i + \sum_{\substack{j=1,\\ j\neq i}}^{n} a_{ji} + \beta_{a_i}^* S_{i_0} x_3}{-\beta_{s_i} S_{i_0}}$, $x_6 = \frac{(\alpha_{s_i} + \alpha_i)x_5}{\mu_{h_i} + \mu_i + \xi_i}$, $x_7 = \frac{\rho_i x_4 + \xi_i x_6}{\mu_i + \sum_{\substack{j=1,\\ j\neq i}}^{n} e_{ji}}$ and $y_1 = 0$, $y_2 = 1$, $y_3 = \frac{\sigma_i + \mu_i + \sum_{\substack{j=1,\\ j\neq i}}^{n} b_{ji} - (1-\varepsilon_i)\sigma_i y_5}{\varepsilon_i\sigma_i}$,

$y_4 = \frac{\eta_i y_5}{\eta_i+\rho_i+\mu_i}$, $y_5 = -\frac{\beta_{s_i} S_{i_0}}{l_{i_1}}$, $y_6 = 0$, $y_7 = 0$. Then applying Sotomayor's theorem at $(\tilde{E}_0^{(i)}, \beta^*_{a_i})$ we obtain,

$$\Delta_1 = U_2^T \begin{bmatrix} 0 & 0 & 0 & 0 & 0 & 0 & 0 \end{bmatrix}^T = 0,$$

$$\Delta_2 = U_2^T \begin{bmatrix} 0 & 0 & -S_{i_0} & 0 & 0 & 0 & 0 \\ 0 & 0 & S_{i_0} & 0 & 0 & 0 & 0 \\ 0 & 0 & 0 & 0 & 0 & 0 & 0 \\ 0 & 0 & 0 & 0 & 0 & 0 & 0 \\ 0 & 0 & 0 & 0 & 0 & 0 & 0 \\ 0 & 0 & 0 & 0 & 0 & 0 & 0 \\ 0 & 0 & 0 & 0 & 0 & 0 & 0 \end{bmatrix} U_1 = y_2 x_3 S_{i_0} = \frac{\varepsilon_i \sigma_i \Lambda_i}{(\mu_i + \gamma_i + \theta_i + \sum_{\substack{j=1,\\ j\neq i}}^n c_{ji})(\mu_i + \sum_{\substack{j=1,\\ j\neq i}}^n a_{ji})} > 0,$$

$$\begin{aligned}\Delta_3 &= U_2^T \left(\begin{bmatrix} 2m\beta^*_{a_i} S_{i_0} \\ -2m\beta^*_{a_i} S_{i_0} \\ 0 \\ 0 \\ 0 \\ 0 \\ 0 \end{bmatrix} x_3^2 + \begin{bmatrix} 2m\beta_{s_i} S_{i_0} \\ -2m\beta_{s_i} S_{i_0} \\ 0 \\ 0 \\ 2\alpha_i\gamma_i \\ -2\alpha_i\gamma_i \\ 0 \end{bmatrix} x_5^2 + \begin{bmatrix} -\beta_{s_i} \\ \beta_{s_i} \\ 0 \\ 0 \\ 0 \\ 0 \\ 0 \end{bmatrix} 2x_1x_5 + \begin{bmatrix} -\beta^*_{a_i} \\ \beta^*_{a_i} \\ 0 \\ 0 \\ 0 \\ 0 \\ 0 \end{bmatrix} 2x_1x_3 \right), \\ &= -2m(\beta^*_{a_i} x_3^2 + \beta_{s_i} x_5^2) S_{i_0} + 2(\beta_{s_i} x_5 + \beta^*_{a_i} x_3) + 2\alpha_i \gamma_i x_5^2 y_5.\end{aligned}$$

So $\Delta_3 < 0$ if and only if $\nu_i < \frac{(m_{a_i}\beta^*_{a_i} x_3^2 + m_{s_i}\beta_{s_i} x_5^2) S_{i_0} - (\beta_{s_i} x_5 + \beta^*_{a_i} x_3)}{\alpha_i x_5^2 y_5} = \nu_i^*$. As $\Delta_1 = 0$ and $\Delta_2 > 0$, hence applying the Sotomayor's theorem[7], we note that a backward bifurcation exists if $\nu_i > \nu_i^*$ and a forward (transcritical) bifurcation exists if $\nu_i < \nu_i^*$.

2.4 Parameter estimation

For the purpose of parameter estimation, we considered 3 Indian states, namely, Kerala, Maharashtra, and Tamil Nadu as three patches. Figure 1 shows that three patches, Kerala as patch 1, Maharashtra as patch 2, and Tamil Nadu as patch 3, are connected with equal mobility rates ($p_{12} = p_{21} = p_{23} = p_{32} = p_{13} = p_{31} = 0.001$) for all compartments in each patch.

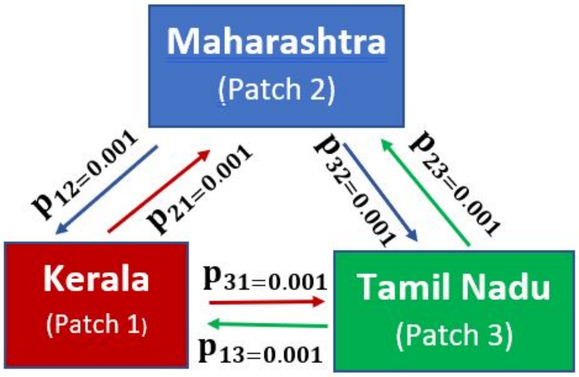

Figure 1. The three-patch model connected with mobility rates p_{12}, p_{21}, p_{13}, p_{31}, p_{23} and p_{32}.

Figure 2 shows the total number of active cases per day for the period of 14 July to 20 August 2021[8] in India, Kerala, Maharastra, and Tamil Nadu, respectively. From all four figures, we observe that in India, including Maharastra and Tamil Nadu total per day active cases is decreasing, but in Kerala, the total number of active cases per day is increasing.

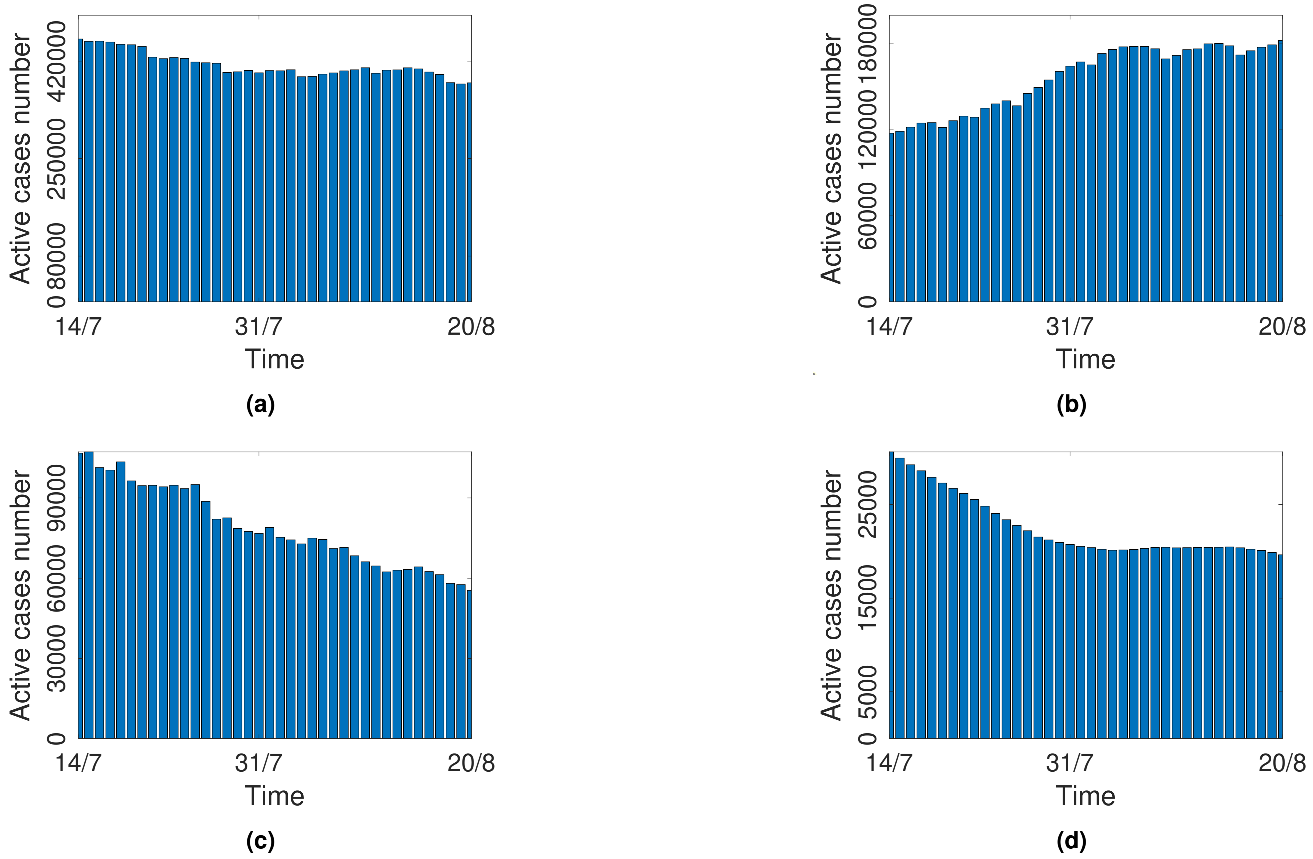

Figure 2. number of active COVID-19 cases in (a) India, (b) Patch 1 (Kerala); (c) Patch 2 (Maharashtra); (d) Patch 3 (Tamil Nadu) from 14th July to 20th August.

References

1. Van den Driessche, P. & Watmough, J. Reproduction numbers and sub-threshold endemic equilibria for compartmental models of disease transmission. *Math. Biosci.* **180**, 29–48 (2002).
2. Meng, L. & Zhu, W. Analysis of covid-19 based on SEIR epidemic models in a multi-patch environment. (2021).
3. La Salle, J. P. *The stability of dynamical systems* (SIAM, 1976).
4. Shuai, Z. & van den Driessche, P. Global stability of infectious disease models using lyapunov functions. *SIAM J. on Appl. Math.* **73**, 1513–1532 (2013).
5. Smith, H. L. & Waltman, P. *The theory of the chemostat: dynamics of microbial competition*, vol. 13 (Cambridge university press, 1995).
6. Rebaza, J. Global stability of a multipatch disease epidemics model. *Chaos, Solitons & Fractals* **120**, 56–61 (2019).
7. Perko, L. *Differential equations and dynamical systems*, vol. 7 (Springer Science & Business Media, 2013).
8. COVID-19, India, data accessed from: https://www.covid19india.org/. on 20th August, 2021.
